# Purpose‐Adaptable Reinforced 3D Hyaluronic‐Acid Based Platform to Study Pathomechanisms of the Central Nervous System

**DOI:** 10.1002/adhm.202505946

**Published:** 2026-02-15

**Authors:** Nicoletta Murenu, Esra Tuerker, Anna‐Lena Wiessler, Jessica Faber, Ievgenii Liashenko, Jeanette Weigelt, Jörg Tessmar, Paul D. Dalton, Sibylle Jablonka, Mateo S. Andrade Mier, Carmen Villmann, Silvia Budday, Natascha Schaefer

**Affiliations:** ^1^ Institute for Clinical Neurobiology University Hospital of Würzburg Würzburg Germany; ^2^ Department of Mechanical Engineering Institute of Continuum Mechanics and Biomechanics Friedrich‐Alexander‐Universität Erlangen‐Nürnberg Fürth Germany; ^3^ Phil and Penny Knight Campus for Accelerating Scientific Impact University of Oregon Eugene Oregon USA; ^4^ Department of Functional Materials in Medicine and Dentistry and Bavarian Polymer Institute University Hospital Würzburg Würzburg Germany

**Keywords:** 3D spinal cord model, 3D disease model, autoimmune disease, hyaluronic acid, inhibitory synapse, laminin

## Abstract

Three‐dimensional (3D) models to study human disease mechanisms have demonstrated that the third dimension is an essential component for neuronal maturation and function. However, 3D neuronal cell culture is challenging due to their ultra‐soft nature and specific extracellular matrix (ECM) organization. This study presents a microfiber reinforcement approach, combining primary mouse spinal cord neurons (SCNs) in a purpose‐adaptable hyaluronic‐acid‐based matrix with melt electrowritten (MEW) frames to study disease mechanisms. The importance of laminins (LNs) is evaluated, which are vital for neuronal adhesion and maturation. Astrocytes (ACs) are mandatory in brain development and function by secretion of signal molecules, maintaining ion homeostasis, clearing of neurotransmitters, and by actively modulating neuronal activity. Three combinations are compared (i) isolated primary SCN, (ii) SCN with ACs (SCN‐AC) and (iii) SCN‐AC and LNs (SCN‐AC‐LN). Multimodal analysis by comparing protein expression, dendrite length, complex mechanical properties, and network functionality via Ca^2+^‐imaging allows validation of structural and functional neuronal network development. The suitability of the 3D model to study pathomechanisms is demonstrated for Stiff Person Syndrome (SPS). For the first time, functional impairment of spinal cord neurons by patient‐derived autoantibodies characteristic for SPS is recapitulated in a 3D spinal cord model platform adaptable to other disease types.

## Graphical abstract

Platform system to create biofabricated 3D spinal cord tissue models: Combining high resolution PCL fiber placement, a customized, hyaluronic acid‐based hydrogel, two cell types (spinal cord neurons and astrocytes) together with three distinct laminin isoforms allow the formation of functional cell–cell network interactions. These tailored 3D spinal cord models are applicable and methodological accessible to study disease pathomechanisms.

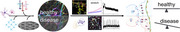

## 1 Introduction

The central nervous system (CNS) demonstrates a unique complexity and hierarchical nature, where the interaction between surrounding molecular and cellular microenvironment plays a central role in maintaining homeostasis and supporting physiological function [1]. For example, the extracellular matrix (ECM) is a specialized, ultra‐soft structure that provides essential biochemical and mechanical support for neuronal survival, differentiation, and synaptic connectivity [2].

As a specialized part of the CNS, the spinal cord is crucial for regulating sensory and motor functions in the human body. Proper motor control and sensorimotor integration critically depend on a precise balance of excitatory and inhibitory signals [3]. Imbalances of those processes underlie neurodegenerative disorders, e.g. motoneuron diseases like amyotrophic lateral sclerosis (ALS), spinal muscular atrophy (SMN) or complex neurological diseases such as stiff baby or stiff person syndrome (SPS). While there is no cure, therapy concentrates on symptomatic improvements, even the molecular mechanisms of those diseases are only partially understood [4, 5].

In vitro two‐dimensional (2D) neuronal culture models have helped to study molecular pathomechanisms of spinal cord diseases [4, 5]. In example, stiff baby syndrome associated with motoneurons dysfunction is caused by mutations in genes encoding inhibitory glycine receptors (GlyRs) or associated proteins of the GlyR complex [5, 6, 7]. However, 2D cellular models failed to fully explain the underlying pathology. This could be due to the fact that neurons within 2D cell culture systems grow in monolayers and interact more with the substrate experiencing a supra‐physiological stiffness, including a limited number of cell‐cell contacts that are not present in native tissue [8, 9]. In SPS, or its severe form progressive encephalomyelitis with rigidity and myoclonus (PERM), autoantibodies target GlyRs and disturb the inhibitory control of spinal cord circuits at the structural and functional level [10, 11]. Moreover, patients with SPS differ in their symptoms and therapeutic responses. In vivo studies using animal models to investigate neuron‐specific dysregulations are challenged by the inaccessibility of either individual cells within the dense nervous tissue or the challenge of conducting functional readouts like Ca^2+^‐imaging in animal models [12]. In addition, these models are costly, ethically controversial, and often unsuitable for studying rare diseases.

Hence, there is a clear need for three‐dimensional (3D) in vitro models that more faithfully represent the neuronal circuitry found in the spinal cord.

Numerous studies have cast or bioprinted 3D spinal cord models with controlled cytoarchitecture, providing advanced platforms to study the molecular pathophysiology of the spinal cord or to develop further therapeutic strategies [1], including cell biology‐based models like spheroids and organoids [13, 14, 15]. All studies share a common bottleneck: the soft nature of spinal cord tissue is difficult to recapitulate, requiring structural reinforcements for those 3D models [16]. Moreover, precise spatial organization within multimaterial scaffolds is critical, together with bioink composition and mechanical properties to strongly influence cell viability, differentiation, and fate of neural cells. Multicellular and multimaterial approaches, including channel‐guided printing and intrascaffold cell assembly, have been shown to promote directional axon growth, heterogeneous mechanical properties, and maturation into functional neural networks. Furthermore, scaffold geometry and biochemical gradients play a key role in guiding region‐specific neurogenesis and enhancing physiological relevance of spinal cord constructs [1].

However, although highly functional, these models lack certain spinal cord ECM molecule compositions and therefore, cannot fully mimic spinal tissue mechanics, leading to aberrant ECM‐specific signaling. Both, the molecular microenvironment of ECM molecules and mechanical characteristics represent key factors influencing neuronal growth and network function [17, 18, 19, 20]. Here, hydrogel‐based 3D models harbor the advantage of precise control over ECM composition and with this, also mechanical characteristics. Such tunability provides a reproducible system for modeling 3D spinal cord physiology and pathology, making it particularly valuable for the use as disease model systems.

We previously initialized a 3D spinal cord model design using Matrigel as a commercially available hydrogel providing ECM [16]. This study demonstrated the importance of the third dimension for neuronal models, showing that onset of protein expression was earlier compared to 2D cultures. Moreover, functional activity starts earlier, demonstrating maturation of neuronal network activity [16]. Although Matrigel seems to resemble a matrix with excellent support of many ECM components, its batch‐to‐batch variations and origin from a mouse sarcoma leave this matrix not suitable for human application [21]. Hyaluronic acid is a major component of the CNS ECM. An acrylated polyethylene glycol (PEGAcr) crosslinked thiolated hyaluronic acid (HA‐SH)‐based hydrogel has been shown to be an appropriate matrix for cortical applications [22, 23]. This system more faithfully recapitulates the ECM of neuronal tissue, which is predominantly composed of an amorphous gel that is rich in glycosaminoglycans such as hyaluronic acid [2, 24]. Specifically in the spinal cord, members of the laminin (LN) superfamily are essential ECM glycoproteins necessary for the proper maturation and function of especially motoneurons [25, 26]. LNs are crucial for spinal cord neurons, promoting cell adhesion, neurite outgrowth, and synapse formation, and thus playing a vital role in the development of proper neuronal networks. LNs consist of three chains (α, β, γ), with each chain contributing to their specificity and nomenclature. In particular, LN‐111 (α1, β1, γ1) and LN‐211 (α2, β1, γ1) are expressed during embryonic development, promoting the axonal elongation of spinal cord motoneurons [27]. In contrast, LN‐221 (α2, β2, γ1) is found in the synaptic cleft of the neuromuscular junction (NMJ). Synapse‐specific LN isoforms associate with Ca^2+^ channels in the presynaptic compartment of motoneurons [27, 28, 29]. This association leads to Ca^2+^ channel accumulations causing increased spontaneous Ca^2+^ influx [27]. This influx in turn can be considered as a stop or differentiation signal for motoneurons [30]. Therefore, LNs can be considered as key players for spinal cord network development and are essential for synapse formation.

A further requirement of the selected materials is that they must closely replicate the mechanical properties of the designed effective 3D neuronal tissue model. Cell‐material interactions and cell‐cell interactions play a crucial role in shaping neuronal tissue development but also the mechanical properties [4, 31]. Astrocytes (ACs) are known for supporting neuronal growth and function, and for acting as primary regulators of neuronal homeostasis in the CNS [32, 33, 34]. Co‐cultures of neurons and ACs in vitro has been shown to promote neuron survival and neurite outgrowth [34, 35, 36, 37, 38].

Furthermore, ACs are known to contribute to the mechanical properties of nervous tissue; in general, ACs are softer than neurons [39, 40]. Correlating the stiffness of human brain tissue from different regions with the relative concentration of ACs demonstrated that the higher the number of ACs, the lower the stiffness [41].

Previous studies using HA‐SH hydrogels have been shown that they support the network formation of cortical neurons when combined with Acs [23, 42]. To provide mechanical support for our 3D samples, MEW‐printed supporting structures using polycaprolactone (PCL) have been used. A triangular scaffold geometry enhances ACs growth and organization [43].

This study highlights the essential contribution of ACs and especially of various LNs as essential ECM components for the development and functional connectivity of spinal cord networks in 3D spinal cord models predicated on a HA‐based hydrogel. The hydrogel with the use of PEGAcr crosslinkers has been developed further to serve as a platform to add essential but specific ECM components for the purpose of tissue design. Here, the designed spinal cord 3D model is used for applicability to understand disease mechanisms. For SPS characterized by patient‐derived autoantibodies targeting spinal cord inhibitory receptors, autoantibody‐targeted functional alterations are successfully identified in the 3D spinal cord model. Using this designed platform, a fully functional 3D spinal cord model is biofabricated with easy adaptation options for other diseases and future application as a test platform for specific therapeutic drug development.

## 2 Results and Discussion

### 2.1 Astrocytes and Laminins Support Neuronal Cell Viability

3D neuronal models are challenging due to the ultra‐soft character of native neuronal tissue, the unique acquisition of the surrounding ECM, which needs to be mimicked and to include a sufficient amount of needed co‐cultured cells [1, 16, 31, 44].

As a matrix, we purpose‐adapted HA‐SH crosslinked with linear PEGAcr (6 kDa) and 8‐arm Star PEGAcr (10 kDa) to covalently bind various LN subtypes, ensuring their sustained availability throughout the culture period. This study concentrates on LNs: LN‐111, LN‐211, and LN‐221 which have been previously shown in 2D as important components for motoneuronal maturation, differentiation, and neuronal network activity specifically in the spinal cord [27, 45]. Laminins consist of three chains (α, β, γ). In particular, the Schwann cell‐specific laminin‐111 (α1, β1, γ1) and the laminin‐211 (α2, β1, γ1) are expressed during embryonic development and promote the axonal elongation [27]. The synapse‐specific laminin‐221 (α2, β2, γ1) is found in the synaptic cleft of the neuromuscular endplate. Synapse‐specific LN isoforms comprise β2 chains and associate with the pore‐forming subunits of N‐ and P/Q‐ type calcium channels (Ca_v_) in the presynaptic compartment of motoneurons [27, 28, 30]. This association leads to Ca_v_ accumulation causing increased spontaneous Ca^2+^ influx at axon terminals [27]. In turn, the enhanced spontaneous Ca^2+^ influx in the axonal ending can be considered as a stop of differentiation signal for the motoneuron [30], leading to a stop in axonal elongation and thereby indirectly triggering the presynaptic maturation of the endplate. Therefore, a synaptic laminin‐calcium channel interaction organizes active zones in motor nerve terminals [46]. Interestingly, knocking out the ß2 chain leads to defective endplate maturation in mice [28, 46]. Due to their named important function during motoneuronal development, maturation and function, a combination of LN‐111, LN‐211, and LN‐221 was selected to optimize the molecular microenvironment as best as possible for the designed 3D spinal cord platform.

Additionally, ACs were combined with the 3D mixed spinal cord culture. The addition of ACs improves neuronal survival and neuronal morphology for cortical neurons grown in 3D [22]. Culturing motoneurons in the laminin‐supplemented HA‐SH 3D multimolecular and multicellular microenvironment enabled neuronal survival of up to 21 days, which is in strong contrast to 2D pure motoneuron cultures. In 2D, those motoneurons die around days 5–7 [47]. Long‐term survival is an essential achievement of the designed 3D spinal cord model as (i) neurodevelopmental disorders, e.g. Amyotrophic lateral sclerosis (ALS) or Spinal Muscular Atrophy (SMA) have a postnatal disease onset. Hence, a 3D system to study molecular pathomechanism for longer periods of time is crucial for elucidating the so far unresolved disease pathology. Due to the ultrasoft character of the spinal cord (<100 Pa) reinforcement of neuronal 3D cultures is fundamental to ensure stability and longer cultivation periods. Based on previous experience, we used MEW‐printed scaffolds with a triangular‐shaped geometry (Figure S1) [16, 22, 23, 43]. These scaffolds improved the handleability of the composites for structural and functional characterizations of cell‐matrix and cell‐cell interactions. Moreover, it increases high throughput capabilities and manipulation capacity [42]. The inclusion of MEW scaffolds provides both mechanical and topographical cues that promote the growth of especially glial cells, like ACs. Moreover, it is known that PCL nanofibers can promote the production of ECM proteins of attached ACs [22, 43, 48]. The combination of MEW‐scaffolds, HA hydrogels and ACs could already be shown to generate mature 3D cortical neuronal networks [22, 42, 43, 48, 49].

To compare the identified essential needs for distinct cellular and molecular microenvironments, especially for spinal cord maturation and network formation, we set up three different conditions: (1) Mixed spinal cord neurons (SCN); (2) mixed SCN and ACs (SCN‐AC); (3) Mixed SCN, ACs and additional LN‐111, ‐221 and ‐211 (SCN‐AC‐LN).

**FIGURE 1:**
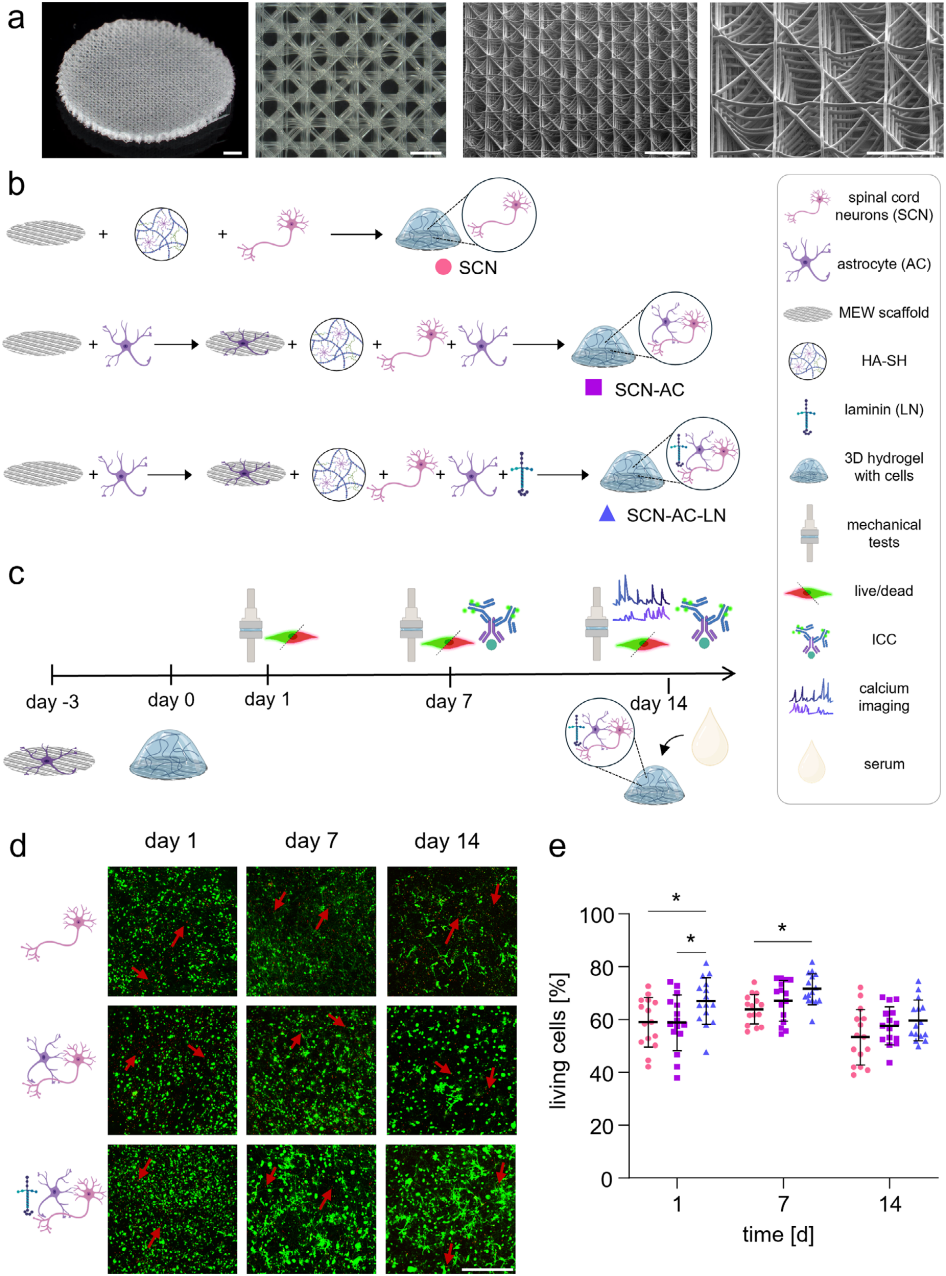
Overview of MEW‐frames, sample conditions, experimental outline, and viability over time. (a) Representative images of MEW‐printed frames from left to right: First: triangular scaffold from 45° angle, scale bar: 1000 µm. Second: optical picture top view, scale bar 100 µm. Third: SEM top view 45° angle, scale bar: 500 µm. Fourth: enlargement of the previous picture, scale bar: 250 µm. (b) Schematic representation of the experimental conditions: isolated spinal cord neurons (SCN), SCN astrocyte co‐culture (SCN‐AC), and SCN‐AC supplemented with LN (SCN‐AC‐LN). (c) Experimental timeline. Primary ACs were seeded onto the PCL scaffold three days prior to the addition of HA‐SH hydrogel containing respective neuronal cells. Model characterization was carried out on days 1, 7, and 14 using live‐dead staining, mechanical testing, immunocytochemistry, and Ca^2+^‐imaging. (d) Viability of spinal cord neurons in HA‐SH was assessed under different culture conditions (SCN, SCN‐AC, and SCN‐AC‐LN). Live‐dead staining was performed using Calcein‐AM (green, live cells) and Ethidium homodimer‐1 (red, dead cells, exemplary cells marked with red arrows). Scale bar: 500 µm. (e) Quantification of cell viability of SCN (● pink), SCN‐AC (■ purple) and SCN‐AC‐LN (▲blue). The analysis was conducted by counting cells in five representative images per experiment (*N* = 3, *n* = 15). Error bars indicate the standard error of the mean. Statistical analysis was conducted using two‐way ANOVA followed by Tukey's *post hoc* test for multiple comparisons. Significance values: ^*^ *p*<0.05.

Sample preparation needed a precise spatial and temporal setup. First, mouse ACs were prepared from p0‐p2 (postnatal days 0 to 2) pups and seeded onto the reinforcing MEW‐printed triangular scaffolds (Figure 1a; Figure S1) for conditions (SCN‐AC and SCN‐AC‐LN). After 3 days, primary mixed SCN were prepared from E13 (embryonic day 13) mice embryos and mixed with HA‐SH (SCN condition) or HA‐SH combined with ACs (SCN‐AC condition) or supplemented with distinct LNs‐111, ‐112, ‐221 in addition to ACs (SCN‐AC‐LN condition). All sample conditions were subsequently plated onto MEW‐scaffolds or MEW‐scaffolds pre‐seeded with ACs for conditions SCN‐AC or SCN‐AC‐LN (Figure 1b).

Characterization of each sample condition was performed at day 1, 7 and 14 for live/dead analysis; day 7 and day 14 for immunocytochemical analysis and mechanical testing. Mechanical testing was also performed without cells at day 1. In addition, as a functional readout, at day 14, neuronal network firing was estimated by Ca^2+^‐imaging measurements (Figure 1c).

In Figure 1d examples of live/dead images from SCN, SCN‐SC and SCN‐AC‐LN samples are depicted. Living cells are marked with Calcein‐AM in green, dead cells in red, stained with Ethidium homodimer‐1 (arrows). All three conditions showed a high rate of survival of cells at all time points. Quantification of live/dead staining revealed slight but significant differences (Figure 1d; Figure S2, Table S1). Cells are marked live or dead without an option to distinguish between SCN and ACs. On day 1, fewer living cells were present in the SCN (58.93%±2.43%) and SCN‐AC (58.8%±2.74%) compared to SCN‐AC‐LN (66.96%±2.27%) conditions. SCN‐AC‐LN samples demonstrated the significantly highest viability of all sample conditions (p*_SCN/SCN‐AC‐LN_ = 0.026, p*_SCN‐AC/SCN‐AC‐LN_ = 0.023). At day 7, a significantly increased viability of cells was only observed for SCN‐AC‐LN compared to SCN condition (SCN: 63.91%±1.45%; SCN‐AC: 67.13%±1.99% and SCN‐AC‐LN: 71.52%±1.53% p*_SCN/SCN‐AC‐LN_ = 0.036). However, at day 14, the number of living cells was similar across all three conditions (SCN: 53.28%±2.70%; SCN‐AC:57.65%±1.86% and SCN‐AC‐LN: 59.64%±1.98%) (Table S1). The quantified percentage of around 60% of living cells after 14 days in culture proves the excellent designed microenvironment for the neuronal composition, especially compared to common 2D cultures, where motoneurons do not survive 7 days in culture [27]. Moreover, it shows the robustness of our platform, demonstrating that neuronal death to a certain extent (up to 30%) does not lead to cellular toxicity in general and is rather compensated by the 3D composite. The observed survival of 60% is comparable to previous studies using cortical neurons in Matrigel or HA‐SH as a hydrogel [42]. In addition, the presented platform is improved by ACs. The positive effect of ACs support is visible when comparing viability at days 7 and 14. The presence of ACs clearly generates a supportive microenvironment for the neuronal cells, most likely due to their secretion of ECM components [32, 33, 34]. These results strengthen our hypothesis that 3D spinal cord cultures require support by LNs and ACs for long‐term survival. However, pure survival of neuronal cells as a criterium for a platform‐system to study disease mechanisms is insufficient. The suitability of a 3D model to study neurological diseases requires an even distribution of spinal cord cell types throughout the 3D sample, but also neuronal maturation determined by dendrite and axon length. Synaptic maturation and development are crucial to allowing neuronal firing within the established network and moreover, are crucial to study disease pathomechanisms.

### 2.2 Impact of Astrocytes and Supplemented Laminins on Dendritic Outgrowth and Mechanical Properties

**FIGURE 2:**
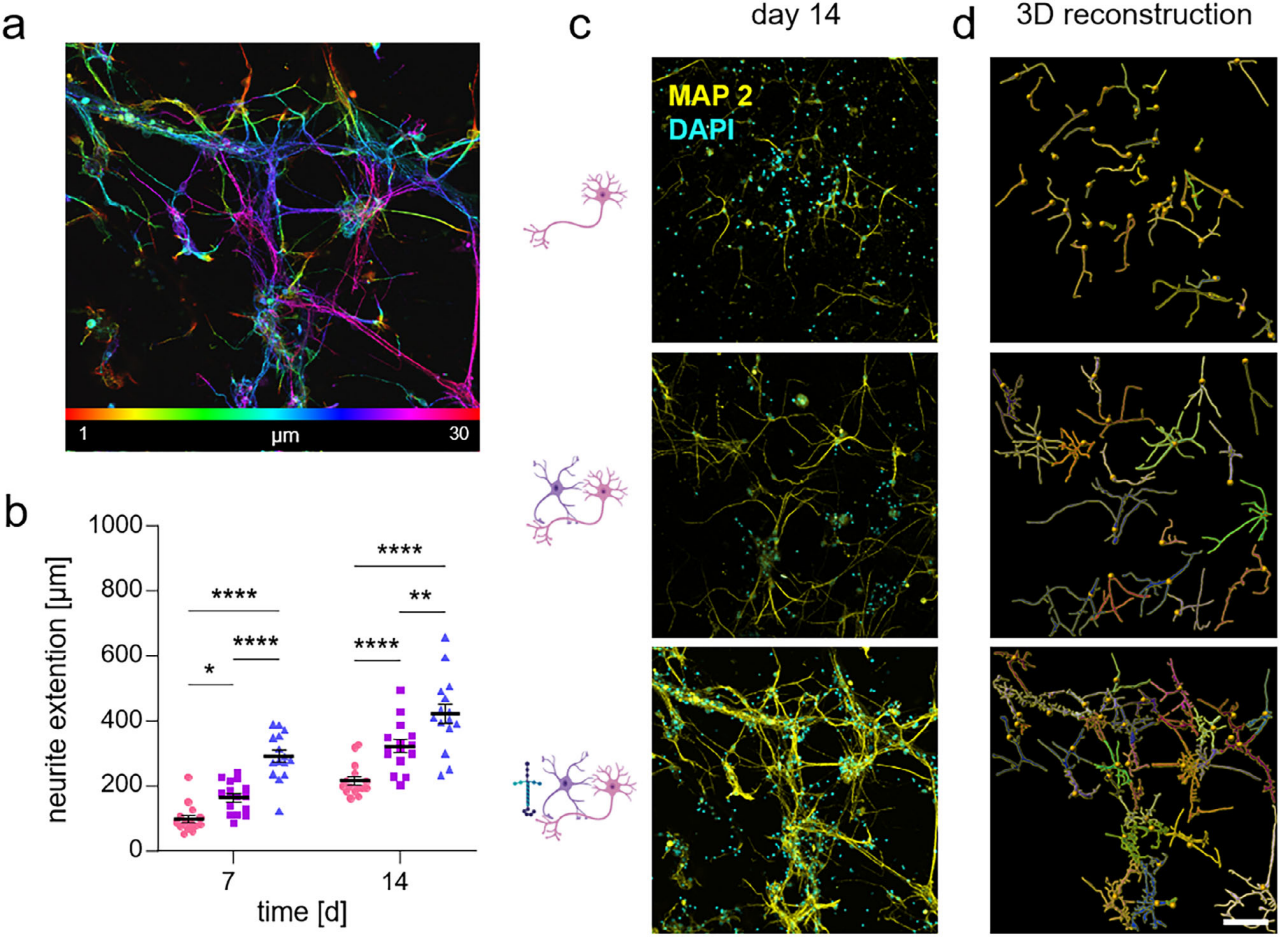
Sample colonization and dendritic outgrowth. (a) Representative Z‐stack of a SCN‐AC‐LN sample at day 14. Color‐coded from red (superficial) to pink (deep), illustrating the spatial distribution of cells along the z‐axis. (b) Quantification of neurite length of SCN (● pink), SCN‐AC (■ purple) and SCN‐AC‐LN (▲ blue). The analysis was performed using five representative images per experiment (*N* = 3, *n* = 15). Error bars represent the standard error of the mean. Statistical analysis was conducted using two‐way ANOVA followed by Tukey's *post hoc* test for multiple comparisons. Significance values: ^*^ *p*<0.05, ^**^ *p*<0.01, ^****^ *p*<0.0001. (c) Immunocytochemistry for neurons using MAP2 (yellow) and nuclei with DAPI (cyan). Scale bar: 100 µm. (d) Visualization of neurite extensions using the filament tracing tool in Imaris. Each color represents a different neuron.

In all three culture conditions and over time, a percentage of around 60% of living cells was detected. To investigate an even distribution of neuronal cells throughout the sample and outgrowth of dendrites being able to form functional networks, Z‐stacks of the culture conditions have been analyzed. An example of the SCN‐AC‐LN condition at day 14 in culture illustrates the spatial and equal distribution of cells along the z‐axis from top to bottom (Figure 2a; Figure S3).

To further compare dendritic outgrowth under different culture conditions, immunocytochemical stainings were performed using the neuronal marker protein microtubule‐associated protein 2 (MAP2) at days 7 and 14. MAP2 is a cytoskeletal marker important for the maturation and differentiation of neurons and correlates with dendrites [50]. MAP2 plays a crucial role in stabilizing the microtubule cytoskeleton, which is essential for neuronal structure and function, but also acts as a binding site for other proteins that influence neuronal development, synaptic plasticity, and signal transduction. At days 7 and 14, SCN‐AC‐LN cultures had significantly longer dendrites compared to SCN‐AC and SCN only cultures (SCN: 97.22µm±11.21; SCN‐AC: 165.2µm±12.8 *p* ^*^_SCN/SCN‐AC_ = 0.093; SCN‐AC‐LN: 290.7µm±18.77 *p* ^****^_SCN/SNC‐AC‐LN_<0.0001, *p* ^****^_SCN‐AC/SNC‐AC‐LN_<0.0001). Even longer dendrites have been observed at day 14 (SCN: 216µm±12.9; SCN‐AC: *p* ^****^_SCN/SCN‐AC_<0.0001; SCN‐AC‐LN: 422.1µm±29.67 *p* ^****^_SCN/SNC‐AC‐LN_ <0.0001, *p* ^*^_SCN‐AC/SNC‐AC‐LN_ = 0.003) (Table S2). In 3D models, dendritic length was analyzed in different hydrogels for cortical neurons [22, 42]. To the best of our knowledge, similar studies have not yet been performed for spinal cord models. Dendrites of spinal cord neurons show longest formations within the SCN‐AC‐LN condition, demonstrating the importance of LNs for spinal cord models and underscoring the relevance the presented platform to be suitable as spinal cord disease model. The molecular pathomechanism in SMA is one disease where reduced axonal outgrowth is a major characteristic [27] and a study using the presented 3D environment could be of high relevance. In 2D, cultured motoneurons survive maximum 7 days in culture and axon lengths reach around 300 µm in length, and only 230 µm under disease conditions [27]. Studying this pathomechanism in 3D using the presented purpose‐adaptable reinforced 3D hyaluronic‐acid based platform has a high potential for the detailed elucidation of the pathomechanisms in a prolonged time window, and with that offers a chance for new pathological insights opening new avenues for therapeutical strategies [51].

Figure 2c exhibits an impression of representative immunocytochemical staining of the SCN, SCN‐AC and SCN‐AC‐LN conditions at day 14 (left), including a 3D reconstruction of neurons (right) (Figure 2d; Video S1) using Imaris Software with which dendritic length was calculated. Note that the neuronal network increases by the provided precise support of carefully chosen LNs that SCNs natively receive. The most‐dense network is visible within sample condition SCN‐AC‐LN. Interestingly, the presence of ACs in SCN‐AC improved dendritic length, but the specific combination of ACs and LNs (LN‐111, LN‐211, LN‐221) exhibits even longer dendrites. LNs are known to be especially important for motoneuron maturation and differentiation. LN‐111 and ‐211 are also known to be essential for axonal outgrowth [27], providing specific support for the purpose of the established platform. Therefore, the presented 3D model has the advantage of temporally stable neuronal cultures, increased temporal cell survival compared to previously used 2D cultures and significantly longer neuronal processes. This enables an application for multiple diseases associated with changes in dendrite length and neuronal morphology.

Taken together, these results demonstrate that both, the addition of ACs to the cellular microenvironment but also the support by ECM components such as distinct LN isotypes are able to mimic the complexity of the spinal cord neuronal network. In addition, to the mentioned cellular and molecular microenvironment, a matching stiffness for neuronal tissue of around 100 Pa is also fundamental for neuronal 3D models for proper maturation of samples [44].

**FIGURE 3:**
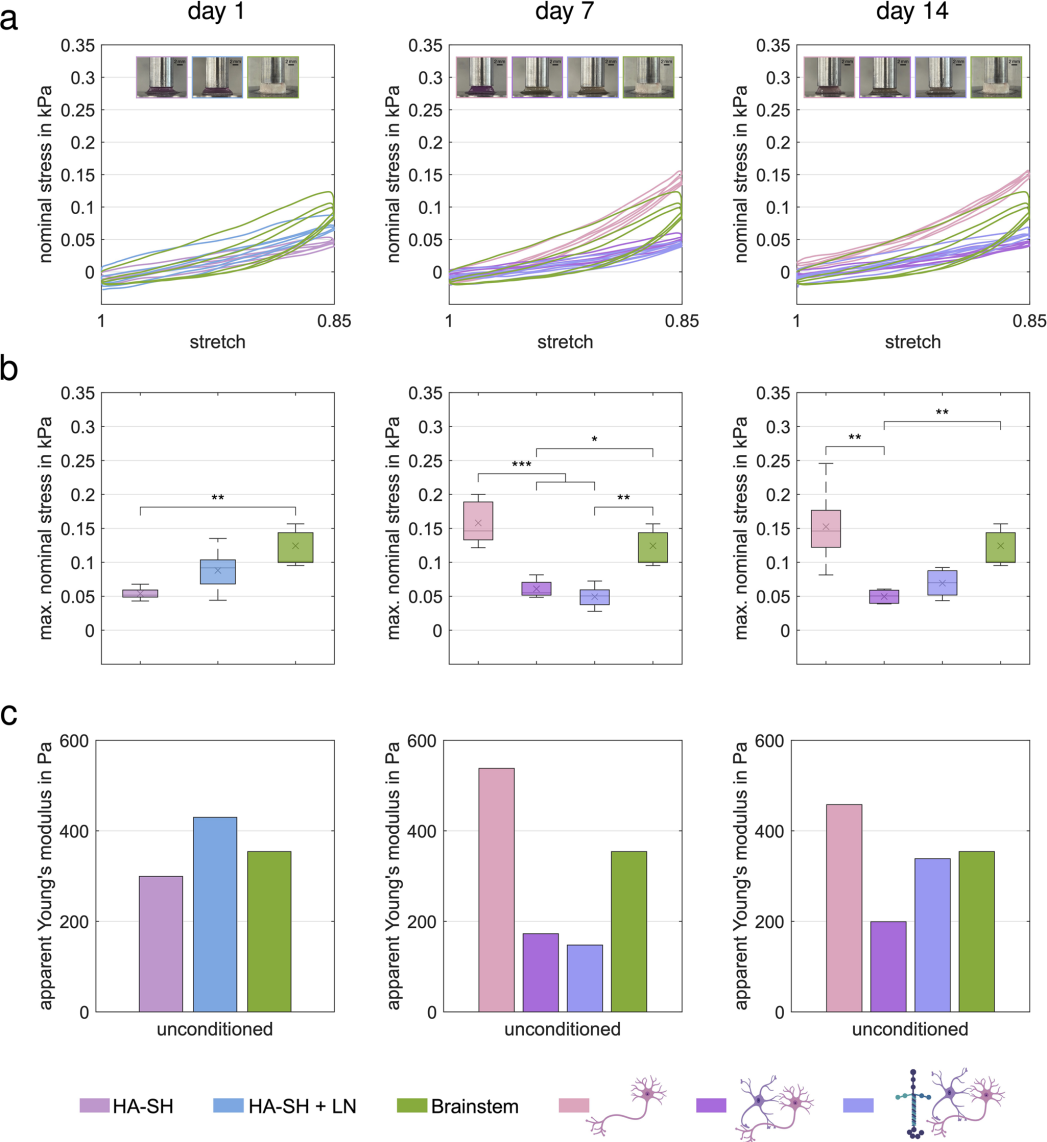
Astrocytes alter mechanical characteristics of the 3D models. (a) Cyclic loading behavior during three loading cycles of unconfined compression up to a maximum strain of 15% at a strain rate of 0.01/s for 0.375% HA‐SH 420 Da with linear and 8‐arm PEGAcr without LN (light pink, *n* = 5) and with LN (light blue, *n* = 5), with SCN (pink, *n* = 5), SCN‐AC (purple, *n* = 5), SCN‐AC‐LN (blue, *n* = 5) and porcine brainstem (green, *n* = 7). The plots include pictures of exemplary samples. Scale bar: 2 mm. (b) Average maximum nominal stresses of the first loading cycle. (c) Corresponding apparent Young's modulus of the first loading cycle from fitting the modified one‐term Ogden model to the experimental cyclic compression data. Significances were calculated using one‐way ANOVA if all samples were normally distributed and Kruskal‐Wallis tests otherwise, followed by Tukey–Kramer tests for multiple comparisons. Significance values: ^*^ *p*<0.05, ^**^ *p*<0.01, ^***^ *p*<0.001.

To test the model for its mechanical properties, cyclic large‐strain mechanical measurements of (cell‐laden) HA‐SH hydrogels and porcine brainstem tissue have been performed. To account for the nonlinear mechanical response of those materials, we used hyperelastic material modeling to determine an apparent Young's modulus, representing the compression stiffness for the specific loading rate applied here. Those measurements and analyses show that the supplementation with LNs does not significantly change the mechanical response of HA‐SH hydrogels without cells on day 1 (maximum nominal stresses in kPa: HA‐SH: 0.05±0.01; HA‐SH+LN: 0.09±0.03; p_HA‐SH/HA‐SH+LN_ = 0.2894; apparent Young's moduli in Pa: HA‐SH: 299; HA‐SH+LN: 430), and with cells on day 7 (maximum nominal stresses in kPa: SCN‐AC: 0.06±0.01; SCN‐AC‐LN: 0.05±0.02 p_SCN‐AC/SCN‐AC‐LN_ = 0.9380; apparent Young's moduli in Pa: SCN‐AC: 173; SCN‐AC‐LN: 148), and day 14 (maximum nominal stresses in kPa: SCN‐AC: 0.05±0.01; SCN‐AC‐LN: 0.07±0.02; p_SCN‐AC/SCN‐AC‐LN_ = 0.8413; apparent Young's moduli in Pa: SCN‐AC: 199; SCN‐AC‐LN: 338) (Figure 3; Figure S4, Tables S3 and S4). This is in line with previous data demonstrating an influence of LNs on the mechanical properties of HA‐SH hydrogels without cells on day 21 using nanoindentation [23]. Furthermore, it agrees well with the study from Spedden et al., where no influence of LNs on the mechanical properties of single murine cortical neurons was detected using atomic force microscopy [52]. Other studies performing cyclic large‐strain measurements on human brain tissue and specimen testing by enzyme‐linked immunosorbent assays showed that LN has little to no relevance for the mechanical properties of most brain regions, including the cortex [53], and that tissue stiffness does not correlate with LN content [41]. Based on these results, it can be concluded that changes in mechanical measurements at later timepoints result solely from the influence of the present cell types (SCN and ACs).

Interestingly, the combination of SCN and ACs in co‐culture (SCN‐AC)—with or without LNs—leads to a significant decrease in stiffness and significantly lower maximum stresses during cyclic loading on day 7 (apparent Young's moduli in Pa: SCN: 538; SCN‐AC: 173; SCN‐AC‐LN: 148; maximum nominal stresses in kPa: SCN: 0.16±0.03; SCN‐AC: 0.06±0.01 *p* ^***^_SCN/SCN‐AC_ = 0.0007; SCN‐AC‐LN: 0.05±0.02 *p* ^***^_SCN/SCN‐AC‐LN_ = 0.0002), and day 14 (apparent Young's moduli in Pa: SCN: 458; SCN‐AC: 199; SCN‐AC‐LN: 338; maximum nominal stresses in kPa: SCN: 0.15±0.06; SCN‐AC: 0.05±0.01 *p* ^**^_SCN/SCN‐AC_ = 0.0084; SCN‐AC‐LN: 0.07±0.02) (Figure 3; Figure S4, Tables S3 and S4). This is in accordance with previous studies on isolated ACs and neurons from murine cerebral cortex [39] and bovine hippocampus [40], which confirm that ACs are softer than neurons. It also agrees with a study correlating the stiffness of human brain tissue from different regions with the relative concentration of different tissue components, including glial fibrillary acidic protein (GFAP) associated with ACs. The analyses exhibited a negative correlation between GFAP concentration and tissue stiffness: the higher the relative concentration of GFAP, the lower the apparent Young's modulus (see Equation 12) [41]. From day 7 to day 14, cell‐laden HA‐SH hydrogels remained stable, and the mechanical properties did not alter significantly. When comparing all 3D models to native brain tissue, we can confirm that the most complex model SCN‐AC‐LN at day 14 exhibits the most similar mechanical stiffness, with an apparent Young's modulus of 338 Pa compared to 354 Pa for porcine brainstem tissue.

In conclusion, we can state that advanced development of the neuronal network within 7 days contributes to the overall mechanical properties, which remain stable within 14 days. Moreover, a stable and suitable environment is important for biological function, mimicking the mechanical characteristics of neuronal tissue in this case spinal cord under physiological conditions.

### 2.3 Astrocytes and Laminins Together Improve Structural and Functional Neural Network Complexity

**FIGURE 4:**
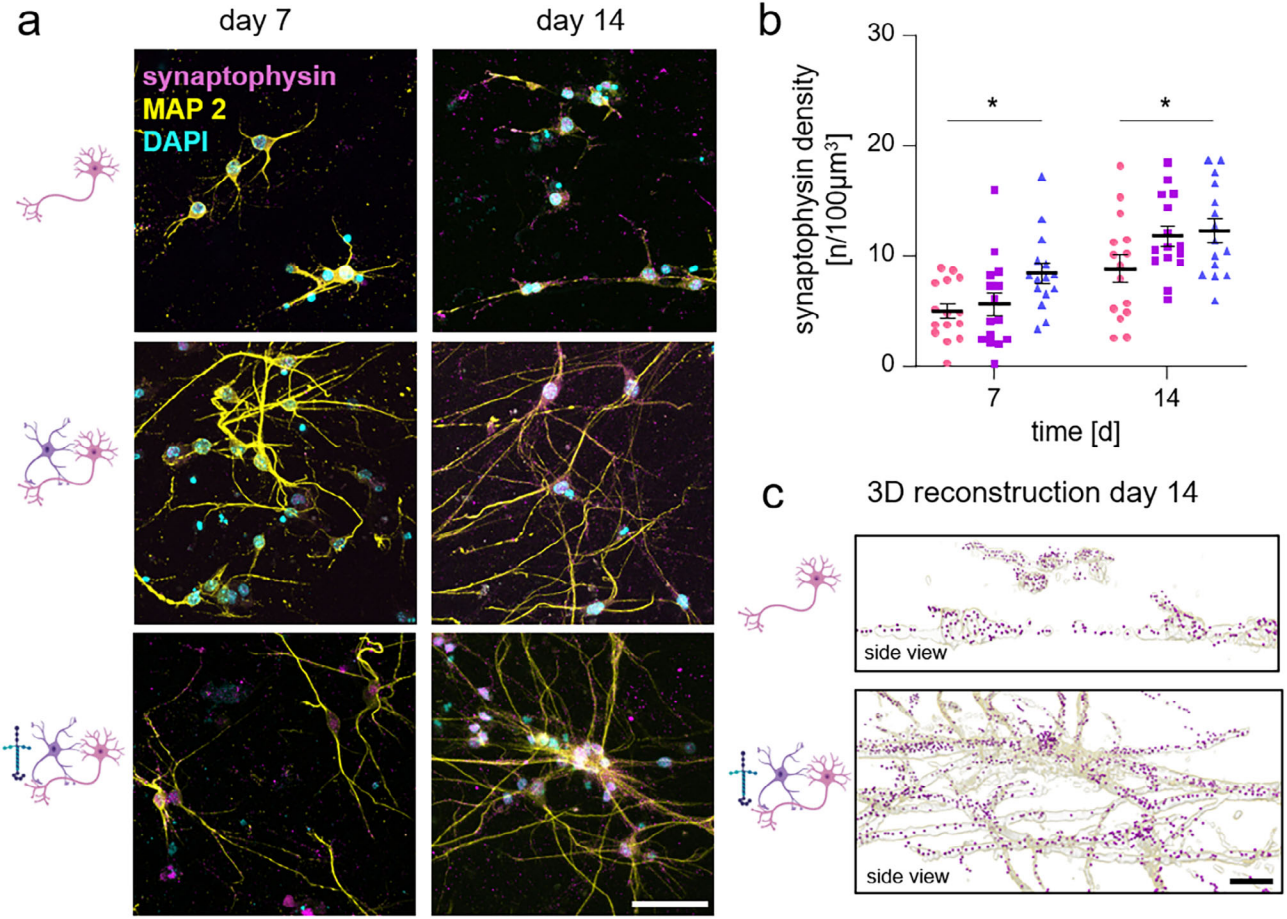
3D spinal cord models form synaptic networks. (a) Representative images showing immunocytochemistry for neurons using MAP2 (yellow), synaptophysin (magenta), and nuclei with DAPI (cyan). Scale bar: 50 µm. (b) Quantification of synaptophysin density of SCN (● pink), SCN‐AC (■ purple) and SCN‐AC‐LN (▲ blue). The analysis was performed over the total MAP2‐positive neuronal volume, using five representative images per experiment (*N* = 3, *n* = 15). Error bars represent the standard error of the mean. Statistical analysis was conducted using two‐way ANOVA followed by Tukey's *post hoc* test. Significance values: ^*^ *p*<0.05. (c) Representative 3D reconstruction showing the synaptophysin (magenta dot) over the entire neuronal volume (yellow) comparing SCN and SCN‐AC‐LN. Original image SCN and SCN‐AC‐LN tilted 90° is displayed in a.

To study neurological disorders in 3D cultures, structural integrity and functionality of the created 3D neuronal networks are fundamental. Structurally, our sample conditions were compared by a combined staining against MAP2, indicating components of the neuronal cytoskeleton, and synaptophysin, a synaptic vesicle protein and marker of synaptic maturation. Representative images of SCN, SCN‐AC and SCN‐AC‐LN samples are shown in Figure 4a. At days 7 and 14, SCN‐AC‐LN cultures displayed a significantly increased synaptophysin density compared to SCN‐AC or SCN only cultures (depicted as numbers of synapses per 100 µm dendrite length; day 7: SCN: 5.06±0.6; SCN‐AC: 5.65±1.06; and SCN‐AC‐LN: 8.51±0.97 *p* ^*^_SCN/SCN‐AC‐LN_ = 0.045 and day 14: SCN: 8.87±1.2; SCN‐AC: 11.83±0.93; and SCN‐AC‐LN:11.79±1.16 *p* ^*^_SCN/SCN‐AC‐LN_ = 0.043) (Figure 4b). Analysis was performed using 3D reconstructions with the Imaris Software and “Spot Detection” to count synaptic density. In Figure 4c representative counts for the culture conditions SCN and SCN‐AC‐LN are depicted. The model comparisons clearly demonstrated that not only the dendritic length is increased, but also the number of synapses (magenta dots) (Table S5 and Video S2). Hence, the formation of a structural neuronal network is dependent on the presence of the third dimension but also on the presence of ACs and the supplementation by LNs as ECM component. LNs are essential for the normal development of the nervous system. ECM in general consists of a mixture of glycoproteins and proteoglycans; important components include collagen, for a structural network, fibronectin and LN for neurite outgrowth [54]. Specifically, the interaction of LN with its receptor integrin largely impacts neurite outgrowth [55]. Downstream signaling after LN bound to its receptor lead to neurite extension and enables neurites to navigate through or along any kind of matrix or other surfaces. [56, 57] This emphasizes the importance of LNs for 3D spinal cord models. Previous publications presented also an importance of ACs for cortical neuronal network formation in a 3D system [42]. For cortical networks a significant increase in synaptic density from day 1 to days 7, 14 and 21 in culture was obvious in 3D, but not in 2D cultures [42].

To further validate the designed 3D spinal cord neuronal network, the functionality of the model was investigated by Ca^2+^‐imaging at 14 days in culture. Ca^2+^‐imaging in general allows the recording of intracellular signals generated by calcium ions that control key functions in all types of neurons. Imaging calcium transients in neurons is particularly important because calcium signals exert highly specific functions in well‐defined cellular subcompartments [58]. For example, in presynaptic terminals, calcium influx triggers exocytosis of neurotransmitter‐containing synaptic vesicles [59]. Postsynaptically, a transient rise of the calcium level in dendritic spines is essential for the induction of activity‐dependent synaptic plasticity [60]. Ca^2+^‐imaging is therefore a highly relevant tool to study disease mechanisms dealing with neuronal excitation [61, 62].

**FIGURE 5:**
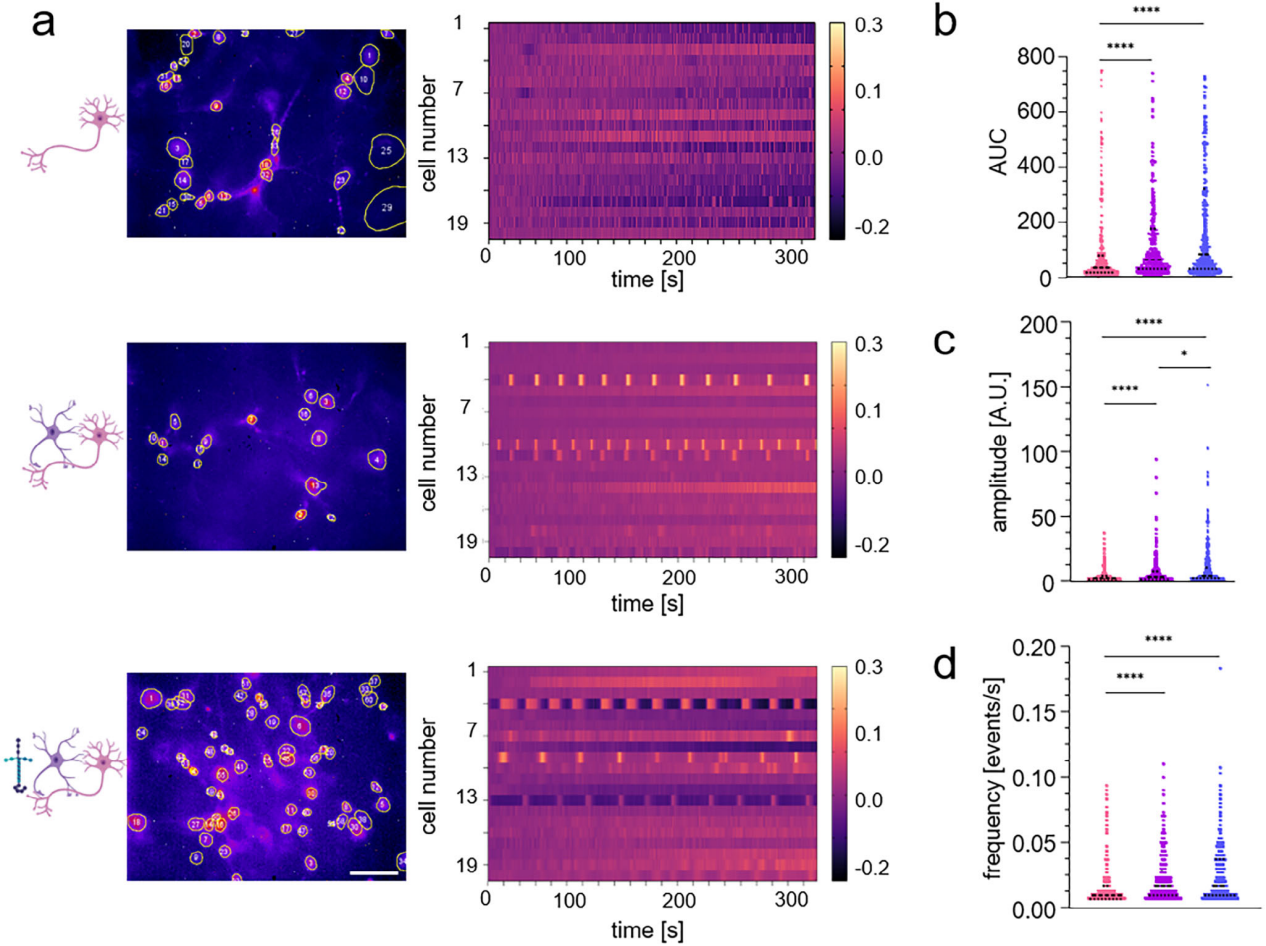
Astrocytes and laminins improve neuronal firing capacities in 3D spinal cord samples. (a) Representative images of Ca^2+^imaging videos performed on day 14 (left, scale bar: 100 µm), heat map representing the portrayed regions of interest (ROI) in a 300s time frame (scale −0.6–0.9 Δ F F−0) (right). (b–d) Activity score of SCN (pink), SCN‐AC (purple) and SCN‐AC‐LN (blue). (*N* = 3, *n* = 280–340cells). AUC (b) amplitude (c) and frequency (d). Statistical analysis was conducted using Kruskal‐Wallis followed by Dunn's *post hoc* test tests for multiple comparisons. Significance values: ^*^ *p*<0.05, ^****^ *p*<0.0001.

Figure 5a exhibits representative images of Ca^2+^‐imaging videos performed on day 14 (left) and a heatmap of ∼20 cells representing the portrayed regions of interest for cultures (SCN, SCN‐AC and SCN‐AC‐LN). The periodically occurring light yellow squares represent Ca^2+^‐oscillations, most likely from ACs as previously described by Janzen et al. [42]. Our analysis focused on the area under the curve (AUC, Figure 5b), amplitude (Figure 5c) and firing frequency (Figure 5d). For all (AUC, amplitude and frequency) analyzed functional characteristics, SCN‐AC‐LN achieved significantly increased functional activity, as compared to SCN and SCN‐AC. Not only the AUC was increased meaning higher and longer events, but samples also showed more events, seen by higher frequencies, demonstrating enhanced neuronal excitation. In addition, higher amplitudes were observed, arguing for increased single events (see Table S6 and Video S3). These data argue for highest spontaneous neuronal activity in the culture condition SCN‐AC‐LN which is most likely a consequence of the detected increased maturation and connectivity (AUC: 354.35±38.48 *p* ^****^_SCN/SCN‐AC‐LN_<0.0001; amplitude (A.U.): 9.74±0.80 *p* ^****^_SCN/SCN‐AC‐LN_<0.0001 *p* ^*^_SCN‐AC/SCN‐AC‐LN_ = 0.036 and frequency (events/s): 0.025±0.0011 *p* ^****^_SCN/SCN‐AC‐LN_<0.0001).

The observed functional differences comparing the sample conditions can be traced back to key functions of LNs and ACs. LNs promote prolonged dendritic outgrowth which in turn allows increased synaptic contact points and denser dendritic networks. Functionally speaking, a denser neuronal network can lead to enhanced excitability by increased numbers of events (frequency) and enlarged amplitudes and AUCs. ACs support the neuronal cultures in providing a super soft mechanical environment and promote neuron survival, neurite outgrowth and healthy neuronal function by secretion of signal molecules, maintaining ion homeostasis, clearing of neurotransmitters, and by actively modulating neuronal activity, leading to increased AUC, frequency and amplitude. From these results, we conclude that SCN‐AC‐LN resembles a mature and functional spinal cord network that allows to study its applicability under disease conditions.

### 2.4 Applicability of the 3D SCN‐AC‐LN Model to Study Disease Mechanisms of Stiff Person Syndrome

**FIGURE 6:**
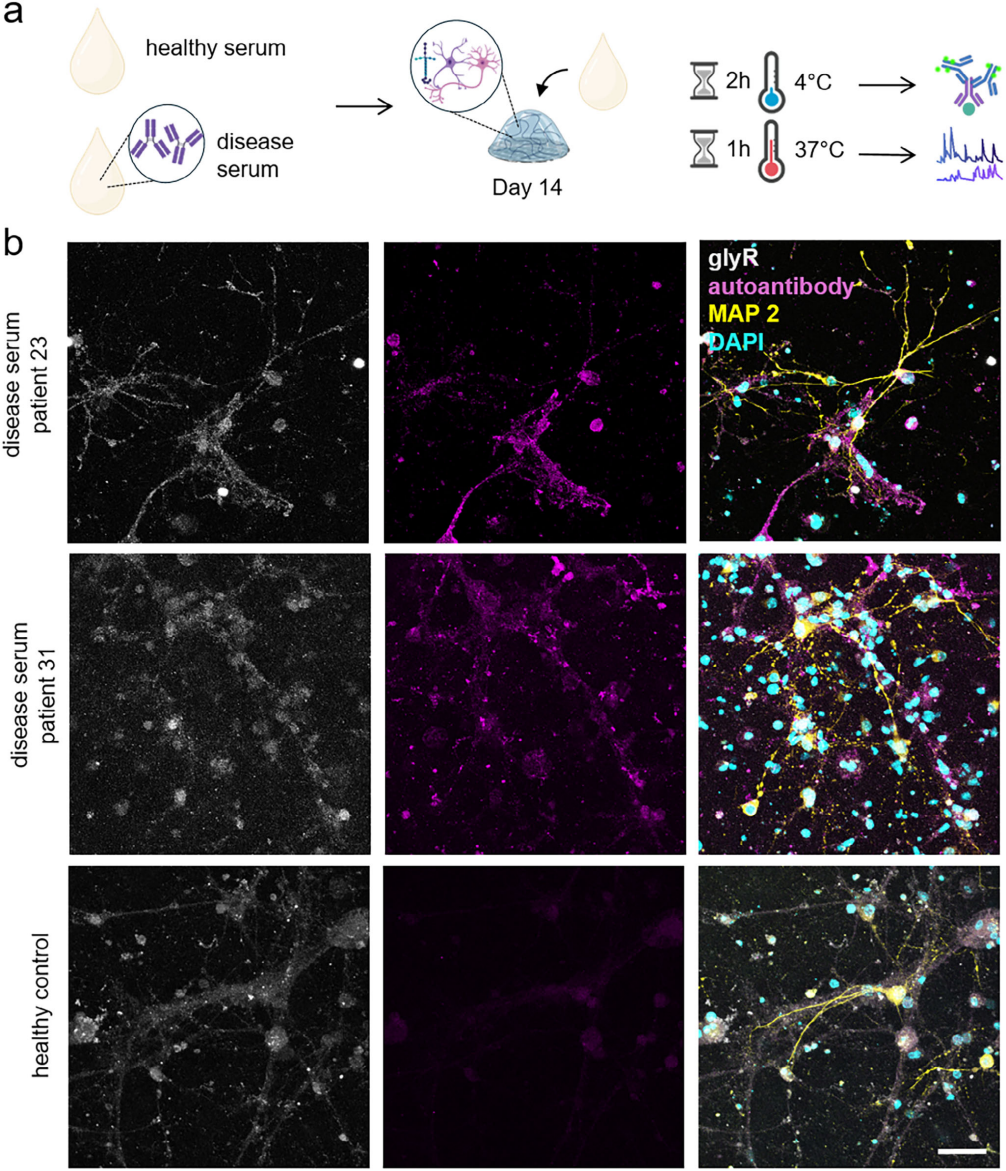
Strong interaction of patient serum with autoantibodies and spinal cord neurons in the 3D SCN‐AC‐LN model. (a) Schematic representation of the experimental workflow. SCN‐AC‐LN 3D co‐cultures were treated with either serum from healthy controls or from patients harboring disease‐associated autoantibodies. Cultures were then analyzed using both Ca^2+^imaging and immunocytochemistry. (b) Representative immunofluorescence images of 3D cultures following serum treatment, showing neuronal structures (MAP2, yellow), GlyRs (gray), patient‐derived autoantibodies (magenta), and nuclei (DAPI, cyan). Scale bar: 50 µm.

To use the designed 3D spinal cord composites as a disease model, we investigated sera from patients with SPS harboring autoantibodies against GlyRs in comparison to a healthy control serum (Figure 6a). GlyR autoantibodies lead to spinal disinhibition resulting in spasms and muscle stiffness via direct functional impairments of GlyR function [10, 63, 64, 65]. One hypothesis in SPS is that reduced hyperpolarization as a consequence of impaired GlyR function in SCNs will eventually lead to hyperexcitability and impaired motoneuron functionality.

To best simulate the disease condition, we selected the SCN‐AC‐LN culture due to its superior performance in previous assays assessing neurite outgrowth, synaptophysin density and Ca^2+^‐imaging. To compare the disease and healthy condition, the consequences of patient‐derived autoantibodies and healthy control serum were investigated (Figure 6a). As a proof of concept, autoantibody binding to GlyRs was controlled by immunocytochemical stainings. Samples were preincubated for 2 h at 4°C. This temperature was chosen to preserve the native distribution of GlyRs and prevent receptor internalization. In contrast, to assess the functional impact of antibody exposure prior to Ca^2+^‐imaging, serum incubation was carried out at 37°C to permit receptor engagement under physiological conditions. Receptor internalization after crosslinking of the proteins by autoantibodies has been described as a hallmark of autoantibody‐mediated diseases [66, 67]. 37°C reflects the body temperature and allows receptor internalization, signaling, and other downstream cellular effects to occur, modeling the functional impact of autoantibody exposure [10, 64, 65].

Immunocytochemistry was conducted investigating serum from different patients and a healthy control serum. The staining pattern from two different patient sera revealed a clear co‐localization between the patient‐derived autoantibodies (magenta) and GlyR clusters (gray) within the MAP2‐positive neuronal network. This co‐localization pattern was absent in cultures treated with control serum (Figure 6b). Again, this result proves that our 3D model can be used to study autoimmune disease.

**FIGURE 7:**
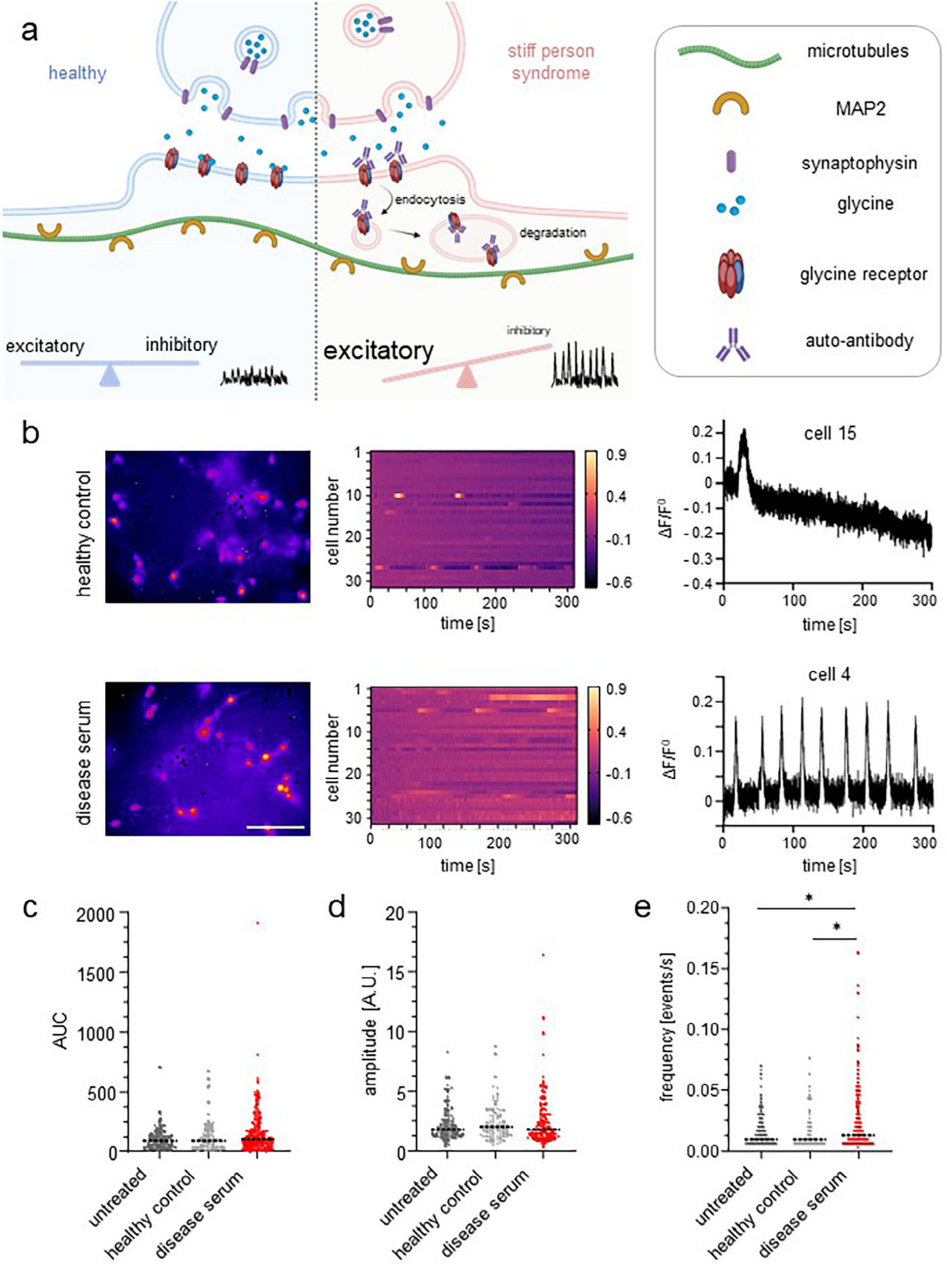
Functional impairment of neuronal function by serum with autoantibodies from SPS patients. (a) Scheme of pre‐ and post‐synaptic regions of inhibitory synapses in healthy and disease conditions. (b) Representative images of Ca^2+^‐imaging videos performed on day 14 (left, scale bar: 100 µm), heat map representing the portrayed regions of interest (ROI) in a 300s time frame (scale −0.6–0.9 Δ F F−0) (middle) and activity trace of one representative cell. (c,d) Activity score of SCN‐AC‐LN cultures untreated (dark gray) and treated with healthy control (light gray) or diseased serum (red) (*N* = 3, *n* = 86–215 cells). AUC (c) amplitude (d) and frequency (e). Statistical analysis was conducted for AUC and amplitude using Kruskal‐Wallis followed by Dunn's *post hoc* test for multiple comparisons. Frequency was conducted using one‐way ANOVA with Holm‐Šídák *post hoc* test for multiple comparison. Significance values: ^*^ *p*<0.05.

Under healthy conditions, glycine is released and binds to GlyRs on the postsynaptic membrane, contributing to fast inhibitory neurotransmission. In the SPS disease context, the presence of GlyR‐specific autoantibodies structurally blocks receptor function [10, 64, 65, 68], leading to a reduction in surface GlyR availability (Figure 7a). This loss of GlyRs diminishes inhibitory glycinergic signaling, leading to network hyperactivity and the pathophysiology observed in autoimmune disorders [63, 64, 65, 68].

To investigate how the loss of inhibitory glycinergic signaling impacts overall neuronal network activity, we next assessed the excitability of the 3D models.

We measured the excitability of 3D cultured SCN via Ca^2+^‐imaging after preincubation with healthy control and disease serum. In addition, untreated neurons were analyzed to exclude effects from the serum itself. All experiments were performed with cultures containing ACs and LN since neuronal activity was highest in those cultures in previous experiments. Exemplary video images from healthy control and disease serum as well as heatmaps of ∼30 cells and fluorescent intensity traces from representative cells are shown in Figure 7b. After disease serum incubation, no differences between the three groups were observed for the AUC and amplitude (Figure 7c,d; Table S7). In contrast, the frequency of spontaneous activity events in SCN‐AC‐LN treated with disease serum was significantly increased compared to incubation with healthy control serum and untreated neurons (untreated: p* = 0.036; healthy control: p* = 0.036; Figure 7e; Table S7 and Video S4). Reduced frequencies of miniature inhibitory postsynaptic potentials (mIPSCs) as a main characteristic of GlyR autoantibodies affecting presynaptic glycine release but also postsynaptic GlyR function have been pointed out by a recent study using brainstem slice recordings in the presence of GlyR autoantibodies [65]. Our observed functional alterations in the spontaneous activity are in line with this. Higher frequencies in Ca^2+^‐imaging point to a generally increased excitability and higher spontaneous firing rate within the spinal cord culture. These are at least to some extent caused by the reduced inhibitory neurotransmission of GlyRs due to autoantibodies leading to reduced mIPSC frequencies, reduced GlyR‐mediated chloride current and thereby an overall reduced inhibition. Additional factors however most likely also contribute to Ca^2+^ signaling effects.

Taken together, our purpose‐designed 3D spinal cord model allowed to recapitulate the disease pathomechanisms for GlyR autoantibody‐mediated SPS. Our data support that the available HA‐based model serves an excellent toolkit being suitable to study other diseases of the CNS and to improve their therapeutic options.

## 3 Conclusion

To achieve suitable 3D biofabricated cellular models is necessary as the importance of the third dimension has been demonstrated to impact neuronal network formation and functionality and thus, impact neurological diseases and their mechanisms. Moreover, 3D biofabricated cellular models offer options for fast high throughput pharmacological testing in combination with the reduction of animal models in line with 3R principles, which represent another challenge in this research field.

**FIGURE 8:**
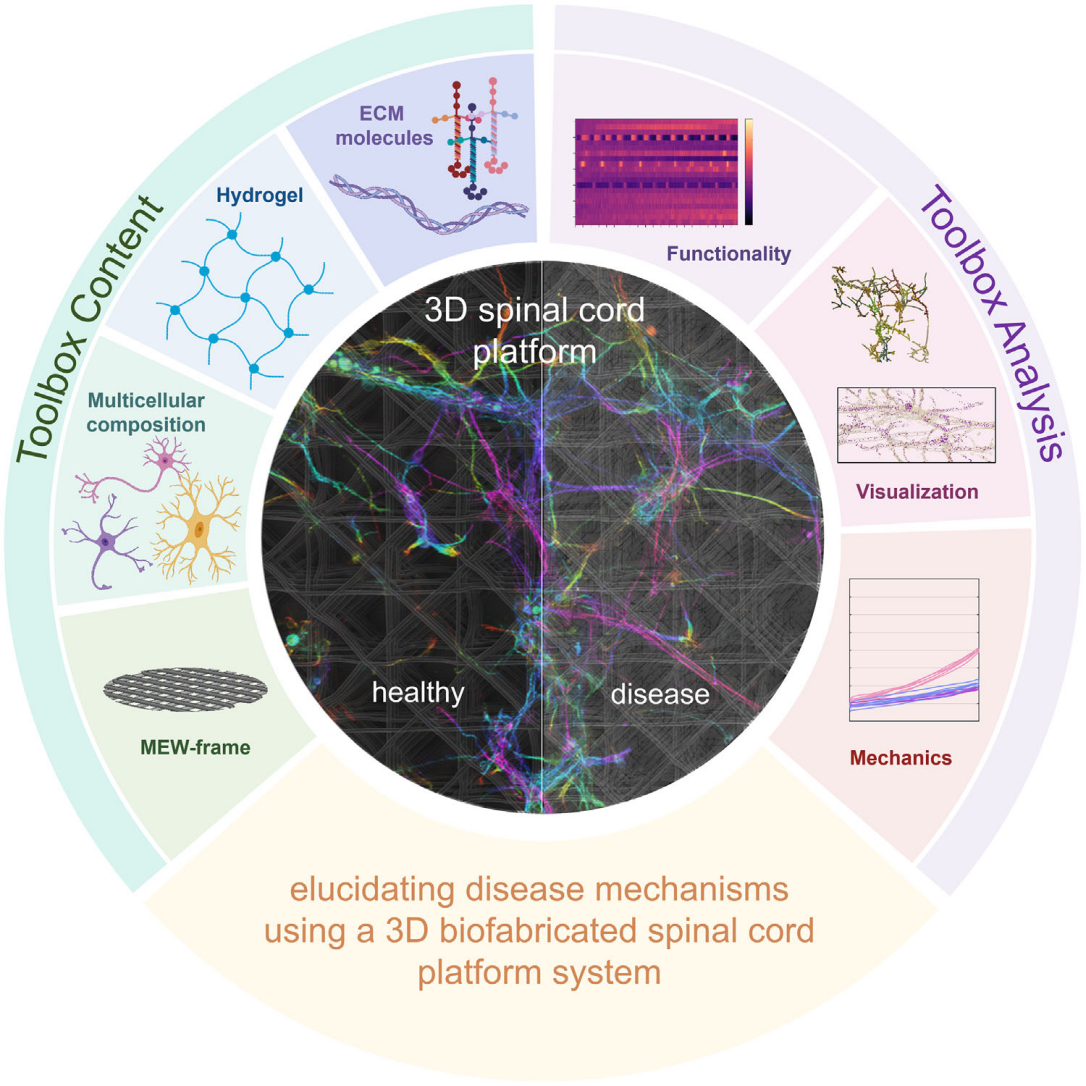
Schematic overview of the easy adaptable 3D spinal cord model. Left area (green background) depicts the key components of the model: ECM molecules, the hydrogel, multicellular composition, and MEW‐frame. Those components represent the toolbox character which can be modified and adapted for other specific applications. The right area (purple background) highlights the multi‐level characterization toolbox. The presented model is validated by mechanical evaluation, visualization via microscopy (allowing structural and morphological analyses), and functional characterizations using Ca^2+^‐imaging. The central part is covered by a reconstruction of the 3D spinal cord network (left side: healthy condition; right: diseased condition) with the MEW‐printed frame visible in the background as grey lines. Together, these versatile approaches (green and purple) enable an easy adaptation of the 3D model (center) to compare healthy and disease‐like 3D conditions, offering a valuable platform to investigate spinal cord physiology and disease mechanisms.

The current study presents a novel platform system creating 3D spinal cord samples to study disease mechanisms in an easy adaptable 3D environment. This ultrasoft 3D spinal cord culture system uses commercially available MEW printed reinforcing scaffolds together with distinct LN isoforms and ACs in a HA‐based hydrogel system. The platform's properties can be easily tuned by adjusting the concentration of HA‐SH and PEGAcr, allowing for the adaptation to other 3D models in health or disease conditions. Therefore, it represents a versatile tool for creating complex biofabricated CNS composites (Figure 8). The toolbox system shows that especially LNs and ACs provide the essential cellular and molecular microenvironment for proper neuronal network formation of the spinal cord. In addition, it facilitates matching stiffness for neuronal tissue of around 100 Pa, which is a prerequisite for precise maturation of 3D neuronal cultures. Our 3D culture system exhibits advanced development of neuronal networks within 7 days and remains mechanically stable up to 14 days. We demonstrate the suitability of the established biofabricated samples to study disease conditions, which in our case is exemplarily shown by the design of an autoimmune disease of the spinal cord. The SCN‐AC‐LN condition to test the patient‐derived serum containing autoantibodies against the inhibitory GlyR revealed a clear co‐localization between the patient‐derived autoantibodies and GlyR clusters in the 3D spinal cord neuronal network. This underlines the proof of concept for our 3D model to study disease conditions. On top of that, we have analyzed the neuronal excitability with the help of Ca^2+^‐imaging in 3D and observed significant hyperexcitability comparing disease and healthy conditions.

Those findings are of major importance in the field of neuronal autoantibody mediated diseases to further identify the disease pathologies. Studies including the third dimension are crucial to elucidate any pathomechanisms, e.g. for other types of spinal cord diseases like SMA, ALS or hyperekplexia.

## 4 Experimental Section/Methods

### 4.1 MEW Scaffolds Printing

MEW Scaffolds were Provided by VivoTex, with Scaffold Design Catalog Number VIS.0004.48.

Circular Scaffolds With a Diameter of 10Mm Were Designed to Consist of four fiber Directions Oriented at 45° Angles to each Other (0°, 45°, 90°, and 135°). Within each Layer, Fibers of 10µm Diameter Were Spaced at 200 µm Intervals. Each fiber Direction Comprised 10 Repeating fiber Layers, Resulting in an Overall Scaffold Thickness of Approximately 150 µm. Medical‐grade Polycaprolactone (PCL, PURAC PC12, Corbion Inc.) Was Used as the Fabrication Material. Scaffolds Were Produced Using a Custom‐built Melt Electrowriting (MEW) Printer. The PCL Melt Was Heated to 75°C and Extruded Through an Electrically Grounded 22G Needle (Nordson 7018260, 6 mm long) That Protruded 1 Mm From the Printhead. The Needle‐to‐substrate Distance Was Fixed at 3Mm, and Scaffolds Were Printed Onto 1Mm Thick Glass Microscopy Slides Placed on a Metal Collector. MEW Parameters Were Optimized to Produce Fibers of 10 µm Diameter, with the Collector Voltage Potential Set to +5.5 kV, Pressure Delivered to the Molten Polymer at 0.3 bar, and Collector Translation Speed at 500Mm/Min.

### 4.2 MEW Scaffolds SEM and Optical Microscopy

As‐printed MEW scaffolds were imaged without further preparation using scanning electron microscopy (SEM, ThermoFisher Apreo 2, USA) using Low Vacuum Secondary Electron Detector (LVSED). Optical images were obtained with Keyence VHX 7000 microscope.

### 4.3 Synthesis of Thiolated Hyaluronic Acid (HA‐SH)

Hyaluronic acid sodium salt (MW = 1–2MDa) was sourced from Biosynth Ltd, Compton, UK. The synthesis followed a previously described method with minor modifications. The molecular weight (MW) and degree of substitution (DS) of HA‐SH were assessed using size exclusion chromatography and nuclear magnetic resonance spectroscopy, respectively (HA‐SH used in this study: MW of 420 kDa and DS of 45%). After freeze‐drying, the final product appeared as white foam.

### 4.4 Synthesis of Polyethylene Glycol‐Acrylates

Polyethylene glycol acrylation (PEGAcr) was conducted for both linear PEG (6 kDa) and 8‐arm Star PEG (10 kDa) following a procedure previously published. PEG (10 g, 1.0 eq.) was first melted at 110°C under high vacuum for 2 h to eliminate residual water. The clear molten PEG was then dissolved in 1L of dry toluene under an argon atmosphere. Dry triethylamine (1.5eq. per PEG diol end group) was added, followed by acrylic acid anhydride (1.5 eq. per functional group). The reaction proceeded at room temperature (RT≈21°C) for 72 h for linear PEG and 4 h for 8‐arm Star PEG. The product was then twice precipitated in cold diethyl ether and repeatedly washed until discoloration was observed. The final purified product, obtained as a white solid, was dried under vacuum at RT.

### 4.5 Primary Cell Culture Astrocyte Isolation

CD‐1 wildtype (strain code 022, Charles River, Sulzfeld, Germany) pups at postnatal day 0–2 (P0‐P2) were used to isolate astrocyte cells. All animal experiments were approved by the local veterinary authority (Veterinäramt der Stadt Würzburg, Germany) and the Ethics Committee of Animal Experiments, i.e., Regierung von Unterfranken, Würzburg, Germany (license no.: FBVVL 568/200‐324/13). The brains of the pups were extracted, and cortices were dissected under binocular. The meninges were removed, and the cortices were collected in cold Dulbecco´s phosphate buffer saline (DPBS 14190144 Gibco, Thermo Fisher Scientific, Massachusetts, US). The tissues were triturated in Dulbecco's Modified Eagle Medium (DMEM, 41 966 029, Gibco New York, US) supplemented with 10% fetal bovine serum (FBS, A5256701 Gibco Thermo Fisher Scientific, US), 2 mM GlutaMAX, and 50U/mL penicillin, and 50 µg/mL streptomycin from Gibco Thermo Fisher Scientific, MA, US) by using glass pipettes with three different tip sizes, which were flamed to remove any sharp edges and filtered through a 70 µm cell strainer (542 070, Greiner Bio‐One, Kremsmünster, Austria). The filtered cell suspension was centrifuged at 1400 rpm for 10 min, and the pellet was triturated with an amount of media, based on the number of pups used, and seeded onto 6 cm cell culture dishes. The cells were cultured at 37°C and 5% CO_2_ for 1 week. Three—four days after seeding, the cells were washed three times with DPBS, and fresh media was added. Three days before SCN isolation, the ACs were detached and seeded onto MEW scaffolds (100.000–150.000 ACs per scaffold).

### 4.6 Primary Cell Culture Spinal Cord Isolation

CD‐1 wildtype mice at embryonic day 12–13 (E12‐13) were used to isolate SCN. The mother mice were euthanized, and E13 stage embryos were harvested. Under binocular, the spinal cords were dissected, cleaned of meninges, and collected in cold DPBS. The collected spinal cord tissues were incubated in 5 mL of trypsin/EDTA (P10‐023500, PAN‐Biotech, Aidenbach, Germany) and 50 uL of DNase I (1 mg/mL; 4 536 282 001, Roche, Mannheim, Germany) for 30 min at 37°C. The reaction was stopped by adding 10% FBS and triturated with a glass pipette, the tip of which was smoothed by flame. The cell suspension was centrifuged at 500 rpm for 10 min. The supernatant was discarded, and the pellet was triturated with 1 mL of neurobasal medium (NB media) (21 103 049, Life Technologies, Waltham, MA, USA) supplemented with 1% B‐27 (17504‐044 Life Technologies, Waltham, MA, USA) and 2 mM GlutaMAX (35 050 038 Gibco, Paisley UK). The trituration step was repeated, centrifuged and SCN were counted.

### 4.7 Scaffold and Hydrogel Preparation

The MEW scaffolds were sterilized in 70% ethanol and subsequently washed three times with phosphate‐buffered saline (PBS, pH 7.4). For samples containing ACs, 150.000 cells were seeded onto the scaffolds in 50 µL of medium 3 days before SCN addition and incubated at 37°C with 5% CO_2_ in DMEM until hydrogel preparation. The lyophilized HA‐SH was sterilized using UV light for 30 min and stored at 4°C until utilized. Stock solutions (15%w/v) of linear PEGAcr and 8‐arm PEGAcr were prepared in PBS and stored at −20°C protected from light. To prepare the hydrogel a stock HA‐SH solutions (1.5% w/v) were dissolved in HEPES buffer (154 mM, pH7.6) and then crosslinked with linear PEGAcr 8‐arm PEGAcr (both 0.75%w/v) in PBS. The solution was incubated at 37 C for 15–20 min before cell seeding. For laminin‐supplemented hydrogels, laminin‐111, laminin‐211 (LN211‐02, Biolamina, Sundbyberg, Sweden) and laminin‐221 (each 30 µg/mL) were used. They were combined with 8‐arm PEGAcr and incubated for 30 min at 37°C in PBS. HA‐SH and linear PEGAcr were added to reach a final volume of 75 µL per sample. After 20 min incubation at 37°C, 450.000 SCN (SCN only condition), 450.000 SCN and 150.000 Acs (SCN‐AC and SCN‐AC‐LN condition) were combined with the hydrogel (with or without LNs), following seeding onto the scaffolds. Following a 30 min incubation at 37°C with 5% CO_2_, 1.5 mL of NB media was added. Since neuronal cells only sparsely adhere onto uncoated glass coverslips, resembling the SCN and SCN‐AC condition [69], no 2D experiments were conducted.

### 4.8 Cell Viability

The viability of SCN and ACs in 3D cultures was evaluated on days 1, 7, and 14 post‐seeding. 3D samples were incubated at room temperature for 30 min with Calcein‐AM (1:500, green/living cells; Thermo Fisher Scientific, USA) and ethidium homodimer (1:1000, red/dead cells; Sigma–Aldrich, USA) diluted in PBS (pH7.4). Samples were imaged using a confocal microscope (Olympus IX81, Japan), and the live/dead ratio was analyzed using the Spots function in Imaris Software 10.4 (Oxford Instrumentals, UK). Three biological replicates (*N* = 3) were performed, culturing two scaffolds per experiment. A total of five image stacks from two scaffolds were analyzed (*n* = 15).

### 4.9 Immunocytochemistry

Samples were washed with PBS (pH 7.4) and fixed with 2% paraformaldehyde for 10 min. Cells were then blocked and permeabilized using 0.2% Triton X‐100 and 5% goat serum in PBS. Primary antibodies, Anti‐MAP2 and Anti‐Synaptophysin (both 1:500; Merck Millipore, USA) were applied overnight at 4°C. Following washing, cells were incubated for 45 min with secondary antibodies Alexa488‐goat‐anti‐mouse and Cy3‐goat‐anti‐rabbit (both 1:500; Dianova, Germany). Finally, the nuclei were stained with DAPI (1:5000, D3571 Invitrogen, Canada) for 10 min.

To assess the suitability of the model as disease model, the constructs were incubated with a 1:100 dilution of patient and healthy control serum and incubated for 2 h at 4°C. Following they were washed with PBS, fixed with 4% PFA/sucrose for 15 min (2D) or 2% PFA/sucrose for 10 min (3D), followed by blocking with 5% goat serum for 30 min. For staining of intracellular proteins, 0.1% Triton‐X100 was added. Cells were incubated for 1 h with primary antibodies MAP2 (1:500) and GlyR α1 (146 118, 1:500). After gently washing, cells were incubated with secondary antibodies (Dianova, Hamburg Germany) for 1 h (Alexa488 (115‐546‐003), Cy5 (111‐175‐006), Cy3 (111‐165‐003). Finally, the constructs were stained with DAPI for 10 min and then stored in PBS at 4°C.

### 4.10 Neurite Extension and Synaptophysin Density Analysis

Neurite Extension Was Quantified Using the “Filament Tracer” Tool in Imaris Software 10.4 (Oxford Instrumentals, UK). MAP2‐stained Neurons Were Imaged at 20× Magnification to Capture the Full Extent of Neurite Outgrowth. The “Filament Tracer” Tool Was Used to Reconstruct the Neurite Architecture in 3D, and the Mean of the Neurite Length per Image Was Extracted From the Resulting Filament Models

For synaptophysin density analysis, neurons were imaged at 60× magnification to enable high‐resolution detection of synaptic puncta. Neuronal volume was defined by MAP2 staining and segmented using the “Filament Tracer” tool to generate a 3D model. Synaptophysin‐positive puncta were detected using the “Spot Detection” tool, and only those within 2.5 µm of the filament surface were included in the analysis using the “Spots Close to Filament” function. The 2.5 µm distance threshold corresponds to the z‐step interval of the image stack. The total number of synaptophysin puncta was normalized to the MAP2‐positive neuronal volume, and synaptophysin density was expressed as the number of puncta per 100 µm^3^.

### 4.11 Ca^2+^‐Imaging

Neuronal activity was assessed using Ca^2+^‐imaging. Cells were labeled with 5 µM Oregon Green 488 BAPTA‐1AM (5 mM stock in 20% Pluronic F127/DMSO; Thermo Fisher Scientific, USA) for 15 min at 37°C with 5% CO_2_. The imaging solution contained (in ×10^−^^3^M): 119 NaCl, 4.5 KCl, 1 MgCl_2_, 2 CaCl_2_, 1.2 NaH_2_PO_4_, 26 NaHCO_3_, 10 glucose, and 10 HEPES, with pH adjusted to 7.4 using NaOH. Image sequences were captured at 10 Hz using the StreamPix4 software (Norpix, Canada) with a Rolera XR Mono fast 1394 CCD camera (Qimaging, Canada) and a cooled epifluorescent light source for 470 nm (Visitron Systems, Germany). Data analysis was done with NeuroActivity Tool [23].

For the disease model, cultures were incubated with healthy control or disease serum for 2 h at 37°C with 5% CO_2_ prior imaging. Imaging was performed as described above. The Fiji plugin “StarDist” was used to define neuron shaped regions of interest (ROIs) with a threshold of 0.6 [70]. To analyze the fluorescent intensity in A.U. within ROIs calculating calcium activity peaks the tool neuralactivitycubic (parameters: signal‐noise‐ratio: 2.2; Noise‐window‐size: 1000; Minimal‐activity‐counts: 2) was used [71].

### 4.12 Porcine Brains

Freshly harvested porcine brain tissue was collected at the local slaughterhouse (Contifleisch GmbH und Unifleisch GmbH, Erlangen, Germany). To extract samples from the brainstem, the tissue was cut into slices of 3 to 4 mm thickness and transferred to a cutting board. Slices were hydrated with Dulbecco's phosphate‐buffered saline solution (DPBS) and brainstem samples were extracted using a cylindrical punch with a diameter of 9 mm. Each sample immediately tested after extraction to minimize gravity‐induced sample deformation.

### 4.13 Large‐Strain Mechanical Behavior

The HR30 Rheometer (TA Instruments, Newcastle, USA) was used to characterize the mechanical properties of 0.375% HASH 420 kDa with linear and 8‐arm PEGDAcr without and with LNs on day 1, as well as with SCN, co‐cultures of SCN and ACs, and co‐cultures SCN‐ACs‐LN on days 7 and 14 under large strains. Samples (*n* = 5 for each condition and time point, *n* = 7 for porcine brainstem) were assumed to slide along the specimen holder surface resulting in a homogeneous deformation state throughout the whole sample. Cyclic compression tests with three loading cycles up to a maximum strain of 15% at a strain rate of 0.01/s were performed at 37°C. During the tests, samples were submerged in Dulbecco's Modified Eagle's Medium – high glucose 4.5 g L−1 (DMEM, Sigma–Aldrich, UK). For one set of experiments the neuronal culture did not show any functionality in Ca^2+^‐imaging, samples were therefore excluded, also for mechanical testing.

### 4.14 Data Preprocessing

The cyclic compression data of the unconditioned (first cycle) and conditioned (third cycle) material responses of HA‐SH and porcine brainstem were used, and the corresponding loading and unloading curves were averaged to approximate the hyperelastic material responses. For further details on the data preprocessing approach, see our previous study [41]. That work demonstrates that identifying the material parameters by fitting the experimental data of every single sample separately and subsequently averaging those material parameters is not accurate and can lead to nonlinear curves that do not match the experimentally observed mechanical responses.

### 4.15 Hyperelastic Material Modeling

To characterize the mechanical properties of 3D models and porcine brainstem from nonlinear mechanical responses in cyclic compression, the nonlinear equations of continuum mechanics were employed. The deformation map ***φ*** (***X***, *t*) was introduced to maps position ***X*** from the undeformed, unloaded configuration $B_{0}\in R^{3}$ at time *t*_0_ to its new position ***x*** in the deformed, loaded configuration $B_{t}$ at time *t*. The deformation gradient ***F *** (***X***, *t*) = ∇_*X*_ ***φ*** (***X***,*t*) maps undeformed line elements to deformed line elements with their position vectors ***X*** and ***x***, respectively. The principal stretches *λ*_1_, *λ*_2_, *λ*_3_ are defined as the square roots of the eigenvalues of the left and right Cauchy Green strain tensors with ***b*** = ***FF** ^T^* and ***C*** = ***F** ^T^ **F***, where [***F***] = diag{*λ*_1_, *λ*_2_, *λ*_3_} [72].

Assuming homogenous sample deformation during unconfined cyclic compression, the analytical solution for the experiments can be calculated. In uniaxial compression, the principal stretch in loading direction λ_3_ is

(1) $$\lambda_{3}=1+\frac{\Delta z}{h}\;≔\;\lambda$$

with *z*‐displacement Δ*z* and initial sample height *h*. Assuming isotropic, direction‐independent material responses for both 3D models and porcine brainstem [73], *λ*_1_ = *λ*_2_ applies. Together with the assumption of isochoric deformation, where no volumetric changes occur during testing, the incompressibility constraint J=detF=1 yields

(2) $$J=\;detF=\lambda_{1}\;\lambda_{2}\;\lambda_{3}=\lambda_{1}^{2}\;\lambda \overset{\mathrm{def}}{=}1.$$

Then, the principal stretches can be computed to *λ*_1_ = *λ*_2_ = *λ* ^−1/2^ and the resulting deformation gradient ***F*** takes the matrix representation

(3) $$\left[F\right]=\left[\begin{array}{ccc} \lambda^{-1/2} & 0 & 0\\ 0 & \lambda^{-1/2} & 0\\ 0 & 0 & \lambda \end{array}\right]\;$$

Furthermore, the Piola stress *P*_zz_ was introduced and defined as the load *f*_z_ per initial cross‐sectional area *A* of the sample in the undeformed configuration. For incompressible hyperelastic materials, the strain energy function *ψ* is defined as

(4) $$\psi =\psi \left(F\right)-p\left(J-1\right),$$

where the Lagrange multiplier *p* represents the hydrostatic pressure and enforces the incompressibility constraint *J* = det ***F*** = 1. To capture the hyperelastic responses of 3D models and porcine brainstem, the modified one‐term Ogden model strain‐energy function *ψ*_*O*_(***F***) [74] for incompressible isotropic hyperelastic materials was adopted

(5) $$\psi_{O}\left(F\right)=\frac{2\mu }{\alpha^{2}}\;\left[\lambda_{1}^{\alpha }+\lambda_{2}^{\alpha }+\lambda_{3}^{\alpha }-3\right]$$

with the classical shear modulus *μ*, the nonlinearity parameter *α*, and the principal stretches *λ*_1_,*λ*_2_,*λ*_3_. For incompressible hyperelastic materials, the Piola stress ***P***, defined as the derivative of the strain‐energy function *ψ* with respect to the deformation gradient [72], is

(6) $$P^{\psi }=\frac{\partial \psi }{\partial F}=\frac{\partial \psi_{O}\left(F\right)}{\partial F}\;-p\;\frac{\partial J}{\partial F}=\frac{\partial \psi_{O}\left(F\right)}{\partial F}\;-pF^{-T}$$

with

(7) $$\frac{\partial \psi_{O}\left(F\right)}{\partial F}=\sum_{i=1}^{3}\frac{\partial \psi \left(F\right)}{\partial \lambda_{i}}\;n_{i}⊗\;N_{i}=\sum_{i=1}^{3}\frac{2\mu }{\alpha }\lambda_{i}^{\alpha -1}\;$$

where ***n***_***i***_ and ***N***_***i***_ are the eigenvectors of the left and right Cauchy Green strain tensors, respectively. Applying the boundary conditions for uniaxial compression, *P*_xx_ = *P*_yy_ , we calculate the Lagrange multiplier *p* to

(8) $$p=\frac{2\mu }{\alpha }\;\left(\lambda^{-\frac{\alpha }{2}}\right)$$

and the Piola stress in *z*‐direction $P_{zz}^{\psi }$ with *λ*_3_ = *λ* to

(9) $$P_{\mathrm{zz}}^{\psi }=\frac{2\mu }{\alpha }\;\left(\lambda^{\alpha -1}-\lambda^{-\frac{\alpha }{2}-1}\right).$$

Finally, the nonlinear least‐squares (nonlinear data‐fitting) algorithm *lsqnonlin* in MATLAB version R2025b 25.2.0.3055257 (MathWorks, USA) was used to calibrate the classical shear modulus *μ* (constrained by 𝜇 > 0) and the nonlinearity parameter *α* by minimizing the objective function

(10) $$\chi^{2}=\sum_{i=1}^{n}{\left(P_{\mathrm{zz}}-\;P_{\mathrm{zz}}^{\psi }\right)}_{i}^{2}\;$$

where *n* is the number of experimental data points, *P*_zz_ the measured Piola stress and $P_{\mathrm{zz}}^{\psi }$ the predicted Piola stress using the modified one‐term Ogden. The quality of the fitting results, in terms of differences in the root mean squared error (RMSE) and the coefficient of determination (*R* ^2^) between experimental data and model output, was quantified. The apparent Young's modulus *E_app_*, as a measure of stiffness for the particular loading conditions applied here, can be calculated from the classical shear modulus *μ* and the initial Poisson's ratio *ν* through the relation

(11) $$E_{\mathrm{app}}=2\mu \left(1+\nu \right).$$

With the incompressibility constraint, ν = 0.5 applies and the apparent Young's modulus is

(12) $$E_{\mathrm{app}}=2\mu \;\left(1+0.5\right)=3\mu .$$

### 4.16 Statistical Analyses

Statistical analysis for mechanical testing was performed by one‐way ANOVA if all samples were normally distributed and Kruskal‐Wallis tests otherwise, followed by Tukey‐Kramer post‐hoc test for multiple comparisons using Statistics Toolbox in MATLAB version R2025b 25.2.0.3055257 (MathWorks, USA) with ^*^ *p*<0.05, ^**^ *p*<0.01, ^***^ *p*<0.001.

Statistical analysis of Ca^2+^‐imaging was performed using GraphPad Prism, version 10.1.2. Data are represented as mean and individual values/cell. Normality of the data was reviewed by Shapiro‐Wilk normality test (*α* = 0.05). Statistical significance was calculated using a one‐way ANOVA or Kruskal‐Wallis test with ^*^ *p*<0.05, ^**^ *p*<0.01, ^***^ *p*<0.001 and ^****^ *p*<0.0001. All numbers of experiments (*N*), cells (n) and *p*‐values are given in Tables S1–S7.

## Funding

Deutsche Forschungsgemeinschaft (DFG, German Research Foundation) – Project number 326 998 133–TRR 225.

## Ethical Statement

The Use of Patient Material Has Been Approved by the Ethics Committee of the Medical Faculty, University of Würzburg, Germany With the Project on “Glycine Receptor Autoantibodies and Spinal Disinhibition” (20190424‐01).

## Conflicts of Interest

The authors declare no conflicts of interest.

## Supporting information




**Supporting File 1**: adhm70940‐sup‐0001‐SuppMat.docx.

**Supporting File 2**: adhm70940‐sup‐0002‐FigureS1‐S4.zip.

**Supporting File 3**: adhm70940‐sup‐0003‐VideoS1‐S4.zip.

## Data Availability

The data that support the findings of this study are available from the corresponding author upon reasonable request.
