# Supplementary material for: Purpose‐Adaptable Reinforced 3D Hyaluronic‐Acid Based Platform to Study Pathomechanisms of the Central Nervous System
Source: Adv Healthc Mater. 2026 Feb 15;15(17):e05946. doi: 10.1002/adhm.202505946 (PMC13175302; doi:10.1002/adhm.202505946)
Supplement: Supplementary file 1 — Supporting File 1: adhm70940‐sup‐0001‐SuppMat.docx. [file ADHM-15-0-s003.docx]

Supporting Information

**Purpose-adaptable reinforced 3D hyaluronic-acid based platform to study pathomechanisms of the central nervous system**

*Nicoletta Murenu, Esra Tuerker, Anna-Lena Wiessler, Jessica Faber, Ievgenii Liashenko, Jeanette Weigelt, Jörg Tessmar, Paul D. Dalton, Sibylle Jablonka, Mateo S. Andrade Mier, Carmen Villmann, Silvia Budday and Natascha Schaefer**

**Figure S1**


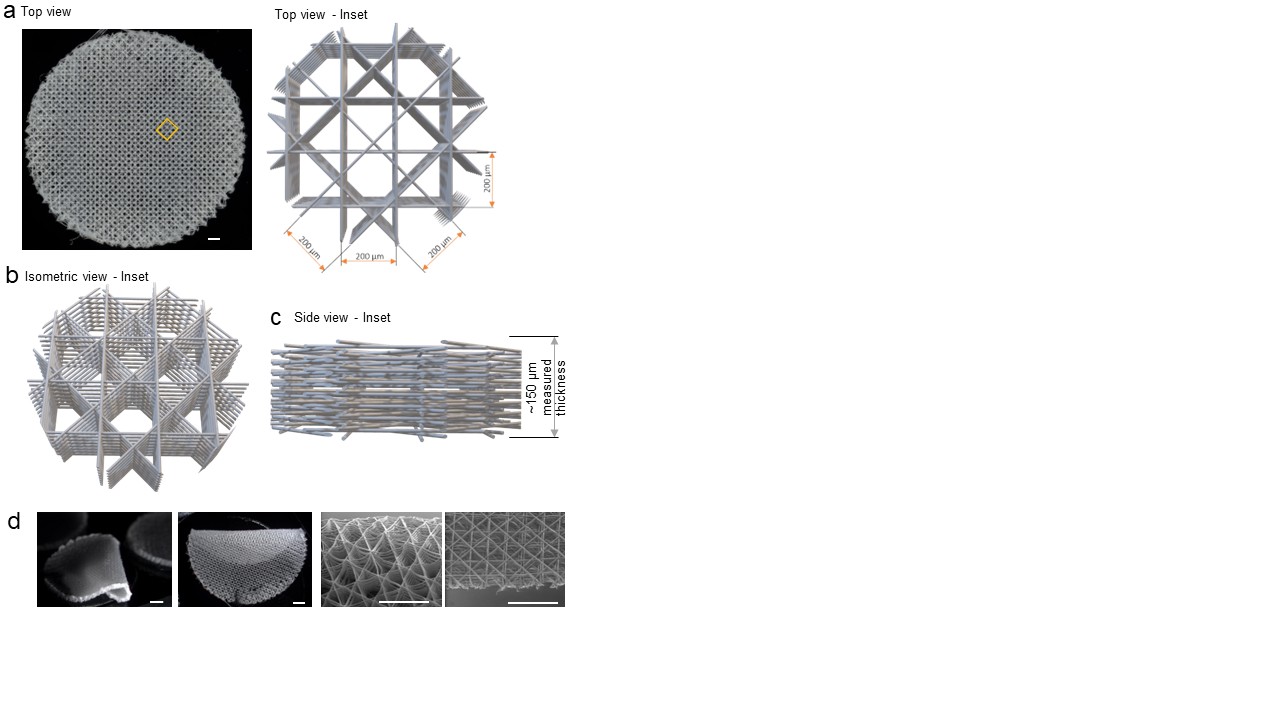


**Figure S1.** MEW-frames.

a) Left: Optical image of the MEW scaffold in top view, with an enlarged inset marked by a yellow square. Scale bar: 500 µm. Right: Top-view 3D model of the enlarged inset, with marked inter-fiber spacing. b) Isometric view 3D model of the enlarged inset marked in (a). c) Side-view 3D model of the enlarged inset marked in (a), with measured scaffold thickness. d) From left to right: Optical images demonstrating the flexibility of the as-printed scaffold when folded. Scale bar: 1 mm. Third image: SEM image showing the structural features of the same scaffold when folded, highlighting the robustness and flexibility of the microfiber scaffold. Scale bar: 500 µm. Fourth image: SEM image of a cut scaffold highlighting the precise alignment of the printed geometry. Scale bar: 500 µm.

**Figure S2**
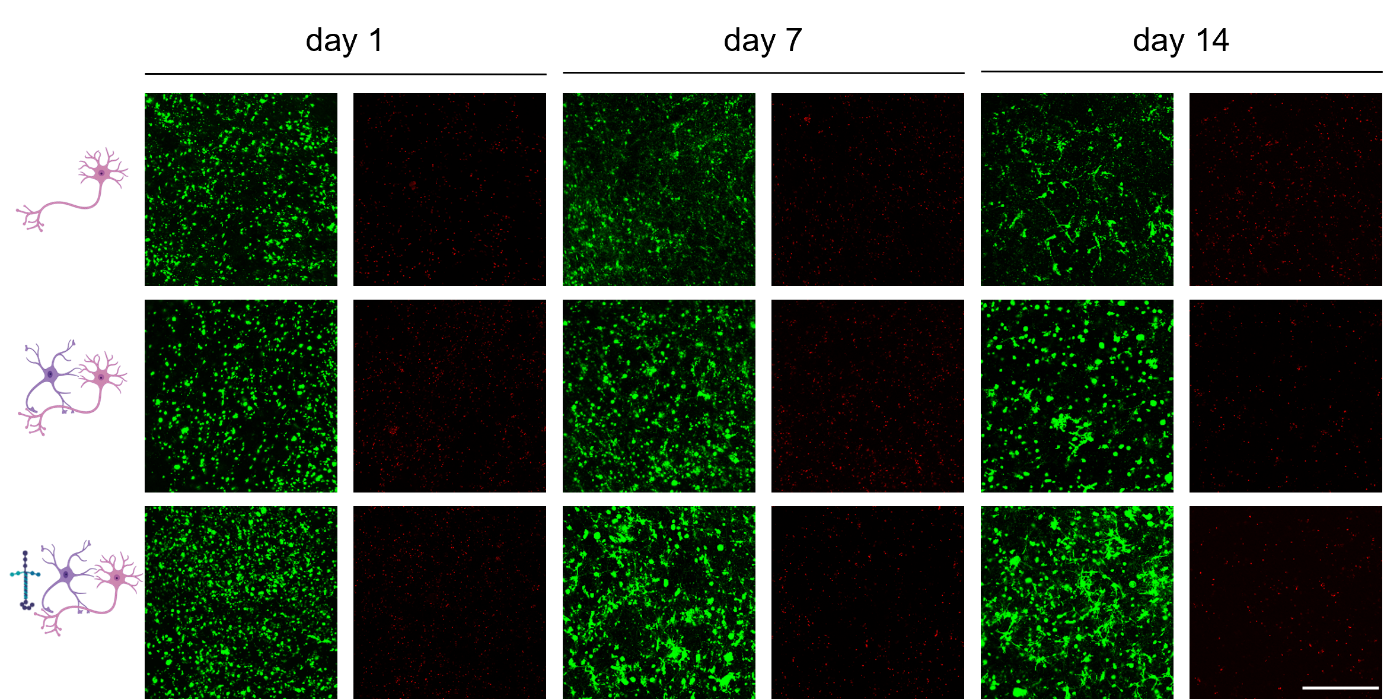


**Figure S2.** Viability assay. Viability of spinal cord neurons in HA-SH was assessed under different culture conditions (SCN top lane, SCN-AC middle lane, and SCN-AC-LN bottom lane). Live-dead staining was performed using Calcein-AM (green, live cells) and Ethidium homodimer-1 (red, dead cells). The scale bar: 500µm.

**Figure S3**


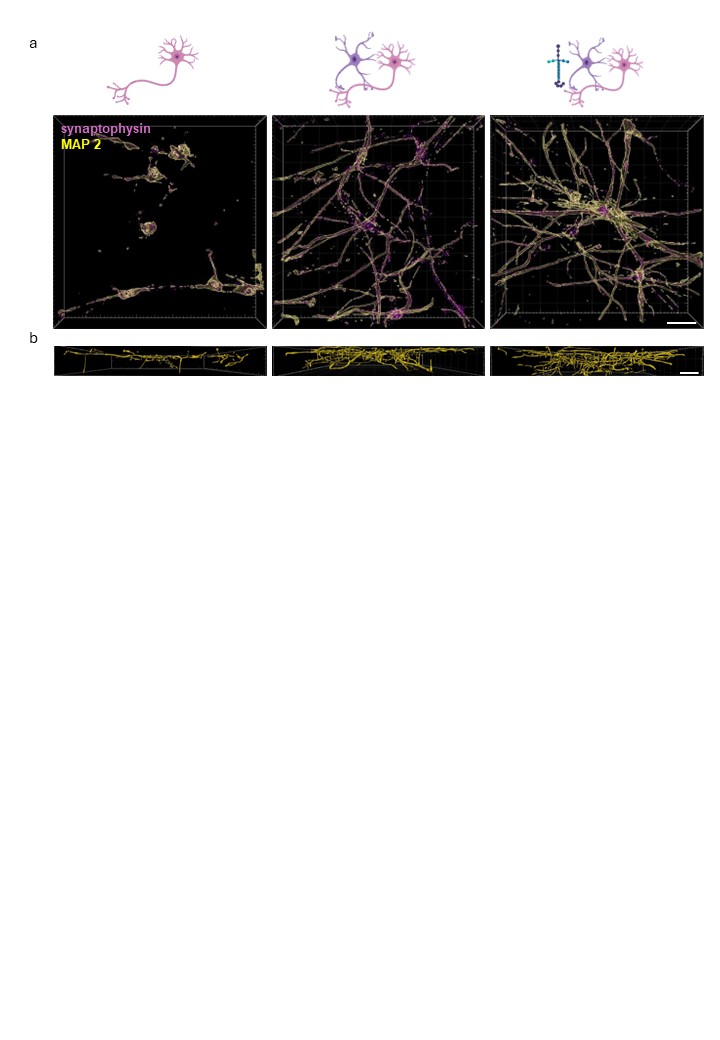


**Figure S3.** 3D reconstruction generated from Z-stack images. a. Representative images from top view SCN left, SCN-AC middle, and SCN-AC-LN right and b. side view of 3D reconstructed model. Scale bar 30µm.

**Figure S4**


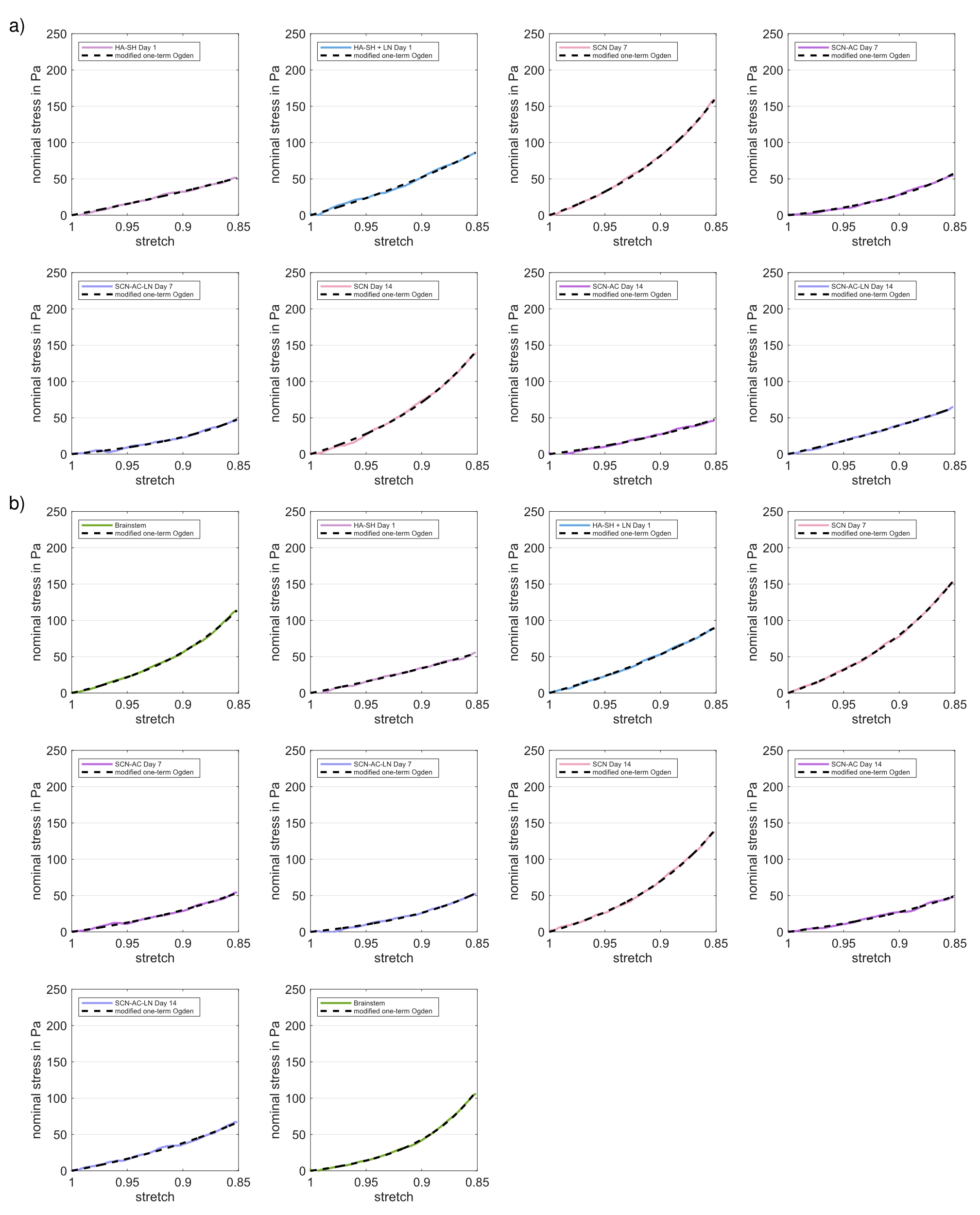


**Figure S4.** Modified one-term Ogden model calibrated with the average a) unconditioned and b) conditioned mechanical responses of 0.375 % HASH 420 Da with linear and 8-arm PEGAcr without LN (n= 5) and with LN (n= 5), with SCN (n= 5), SCN-AC (n= 5), SCN-AC-LN (n= 5) and porcine brainstem (n= 7) in cyclic compression up to a strain of 15 % at a strain rate of 0.01/s.

**Table S1.** Live/dead as the percentage of live cells comparing SCN, SCN-AC and SCN-AC-LN.

|  | day 1 | | day 7 | | day 14 | |  |
| --- | --- | --- | --- | --- | --- | --- | --- |
| live cells [%] | mean±sem | n | mean±sem | n | mean±sem | n | |
| SCN | 58.93±2.43 | 15 | 63.91±1.45 | 15 | 53.28±2.70 | 15 | |
| SCN-AC | 58.8±2.74 | 15 | 67.13±1.99 | 15 | 57.65±1.86 | 15 | |
| SCN-AC-LN | 66.96±2.27 | 15 | 71.52±1.53 | 15 | 59.64±1.98 | 15 | |
|  | **p value** | | **p value** | | **p value** | |  |
| SCN vs. SCN-AC | ns 0.999 | | ns 0.541 | | ns 0.326 | | |
| SCN vs. SCN-AC-LN | *0.026 | | *0.036 | | ns 0.096 | | |
| SCN-AC vs. SCN-AC-LN | *0.023 | | ns 0.324 | | ns 0.791 | | |

Significances were calculated using two-way ANOVA followed by Tukey's post hoc test tests for multiple comparisons.: *p≤0.05; **p≤0.01; ***p≤0.001; ****p≤0.0001; ns = not significant

**Table S2.** Neurite extension comparing SCN, SCN-AC and SCN-AC-LN.

|  | day 7 | | day 14 | |  |
| --- | --- | --- | --- | --- | --- |
| neurite extension [µm] | mean±sem | n | mean±sem | n | |
| SCN | 97.22±11.21 | 15 | 216±12.9 | 15 | |
| SCN-AC | 165.2±12.8 | 15 | 332.9±18.96 | 15 | |
| SCN-AC-LN | 290.7±18.77 | 15 | 422.1±29.67 | 15 | |
|  | **p value** | | **p value** | |  |
| SCN vs. SCN-AC | *0.0293 | | ****<0.0001 | | |
| SCN vs. SCN-AC-LN | ****<0.0001 | | ****<0.0001 | | |
| SCN-AC vs. SCN-AC-LN | ****<0.0001 | | *0.003 | | |

Significances were calculated using two-way ANOVA followed by Tukey's post hoc test tests for multiple comparisons.: *p≤0.05; **p≤0.01; ***p≤0.001; ****p≤0.0001; ns = not significant

**Table S3.** Cyclic compression tests comparing HASH, HA-SH + LN, SCN, SCN-AC, SCN-AC-LN and porcine brainstem.

|  | **day 1** | |  |  |  |
| --- | --- | --- | --- | --- | --- |
| **Maximum nominal stress [kPa]** | mean±sem | n |  |  |  |
| **HA-SH** | 0.05±0.01 | 5 |  |  |  |
| **HA-SH + LN** | 0.09±0.03 | 5 |  |  |  |
| **Brainstem** | 0.12±0.04 | 7 |  |  |  |
|  | **p value** | |  |  |  |
| **HA-SH vs. HA-SH + LN** | ns 0.2894 | |  |  |  |
| **HA-SH vs. Brainstem** | **0.0092 | |  |  |  |
| **HA-SH + LN vs. Brainstem** | ns 0.2043 | |  |  |  |
|  | **day 7** | | **day 14** | |  |
| **Maximum nominal stress [kPa]** | mean±sem | n | mean±sem | n | |
| **SCN** | 0.16±0.03 | 5 | 0.15±0.06 | 5 | |
| **SCN-AC** | 0.06±0.01 | 5 | 0.05±0.01 | 5 | |
| **SCN-AC-LN** | 0.05±0.02 | 5 | 0.07±0.02 | 5 | |
|  | **p value** | | **p value** | |  |
| **SCN vs. SCN-AC** | ***0.0007 | | **0.0084 | | |
| **SCN vs. SCN-AC-LN** | ***0.0002 | | ns 0.0897 | | |
| **SCN vs. Brainstem** | ns 0.2996 | | ns 0.9906 | | |
| **SCN-AC vs. SCN-AC-LN** | ns 0.9380 | | ns 0.8413 | | |
| **SCN-AC vs. Brainstem** | *0.0148 | | **0.0098 | | |
| **SCN-AC-LN vs. Brainstem** | **0.0039 | | ns 0.1167 | | |

Significances were calculated using one-way ANOVA if all samples were normally distributed and Kruskal-Wallis tests otherwise, followed by Tukey's post hoc test tests for multiple comparisons.: *p≤0.05; **p≤0.01; ***p≤0.001; ns = not significant

**Table S4.** Ogden model parameters and apparent Young's moduli E_app

|  | unconditioned | | | | | conditioned | | | | |
| --- | --- | --- | --- | --- | --- | --- | --- | --- | --- | --- |
|  | $\alpha$ | $\mu$ | RMSE | *R^2^* | *E*_app_ | $\alpha$ | $\mu$ | RMSE | *R^2^* | *E*_app_ |
|  |  | in Pa | in Pa |  | in Pa |  | in Pa | in Pa |  | in Pa |
| HA-SH Day 1 | 2.99 | 99.80 | 1.02 | 1.00 | 299 | 0.48 | 98.12 | 1.16 | 0.99 | 294 |
| HA-SH + LN Day 1 | -1.42 | 143.25 | 1.46 | 1.00 | 430 | -2.69 | 139.67 | 0.82 | 1.00 | 419 |
| SCN Day 7 | -7.67 | 179.33 | 0.96 | 1.00 | 538 | -7.56 | 175.83 | 0.99 | 1.00 | 527 |
| SCN-AC Day 7 | -9.11 | 57.56 | 0.97 | 1.00 | 173 | -4.71 | 74.13 | 1.07 | 1.00 | 222 |
| SCN-AC-LN Day 7 | -8.85 | 49.23 | 1.06 | 0.99 | 148 | -8.93 | 54.45 | 1.23 | 0.99 | 163 |
| SCN Day 14 | -8.22 | 152.62 | 1.81 | 1.00 | 458 | -8.51 | 147.80 | 1.08 | 1.00 | 443 |
| SCN-AC Day 14 | -4.67 | 66.40 | 1.29 | 0.99 | 199 | -4.99 | 66.34 | 1.18 | 0.99 | 199 |
| SCN-AC-LN Day 14 | 0.12 | 112.80 | 0.70 | 1.00 | 338 | -3.86 | 96.71 | 1.18 | 1.00 | 290 |
| Brainstem | -8.76 | 118.50 | 0.87 | 1.00 | 354 | -14.31 | 68.60 | 0.73 | 1.00 | 206 |

Nonlinearity parameters (*α*), classical shear moduli (*µ*), root mean square errors (RMSE), coeﬃcients of determination (*R^2^*), and apparent Young’s moduli (*E_app_*) of the unconditioned and conditioned mechanical responses of 0.375 % HASH 420 Da with linear and 8-arm PEGAcr without LN (n= 5) and with LN (n= 5), with SCN (n= 5), SCN-AC (n= 5), SCN-AC-LN (n= 5) and porcine brainstem (n= 7) obtained from fitting the analytical solution of the modified one-term Ogden model to the first and third cycle of cyclic compression data up to a strain of 15 % at a strain rate of 0.01/s.

**Table S5.** Synaptophysin density comparing SCN, SCN-AC and SCN-AC-LN.

|  | day 7 | | day 14 | |  |
| --- | --- | --- | --- | --- | --- |
| synaptophysin density [number per 100µm^3^ neurite] | mean±sem | n | mean±sem | n | |
| SCN | 5.06±0.68 | 15 | 8.87±1.21 | 15 | |
| SCN-AC | 5.65±1.06 | 15 | 11.83±0.93 | 15 | |
| SCN-AC-LN | 8.51±0.97 | 15 | 11.79±1.16 | 15 | |
|  | **p value** | | **p value** | |  |
| SCN vs. SCN-AC | ns 0.906 | | ns 0.095 | | |
| SCN vs. SCN-AC-LN | *0.045 | | *0.043 | | |
| SCN-AC vs. SCN-AC-LN | ns 0.117 | | ns 0.936 | | |

Significances were calculated using two-way ANOVA followed by Tukey's post hoc test tests for multiple comparisons.: *p≤0.05; **p≤0.01; ***p≤0.001; ****p≤0.0001; ns = not significant

**Table S6.** Results of Ca^2+^-imaging comparing SCN, SCN-AC and SCN-AC-LN.

|  | AUC | | relative amplitude | | frequency | |  |
| --- | --- | --- | --- | --- | --- | --- | --- |
| Ca^2+^-imaging | mean [] ± sem | n | mean [A.U.] ± sem | n | mean [event/sec] ± sem | n | |
| SCN | 105.41±11.27 | 337 | 3.80±0.28 | 337 | 0.017±0.0009 | 337 | |
| SCN-AC | 204.79±25.74 | 337 | 6.43±0.55 | 337 | 0.023±0.001 | 337 | |
| SCN-AC-LN | 354.35±38.48 | 372 | 9.75±0.81 | 372 | 0.026±0.0011 | 372 | |
|  | **p value** | | **p value** | | **p value** | |  |
| SCN vs. SCN-AC | ****<0.0001 | | ****<0.0001 | | ****<0.0001 | | |
| SCN vs. SCN-AC-LN | ****<0.0001 | | ****<0.0001 | | ****<0.0001 | | |
| SCN-AC vs. SCN-AC-LN | ns 0.125 | | *0.036 | | ns 0.568 | | |

Significances were calculated using Kruskal-Wallis followed by Dunn´s post hoc test tests for multiple comparisons.: *p≤0.05; **p≤0.01; ***p≤0.001; ****p≤0.0001; ns = not significant

**Table S7.** Results of Ca^2+^-imaging on the SCN-AC-LN sample, comparing untreated, healthy control and disease serum.

|  | AUC | | rel. amplitude | | frequency | |
| --- | --- | --- | --- | --- | --- | --- |
| Ca^2+^- imaging | mean [] ± sem | n | mean [A.U.] ± sem | n | mean [event/sec] ± sem | n |
| untreated | 106.2±8.44 | 118 | 2.2±0.12 | 118 | 0.017±0.0014 | 118 |
| healthy control | 125.6±14 | 86 | 2.3±0.16 | 86 | 0.017±0.007 | 86 |
| disease serum | 149.9±12 | 215 | 2.5±0.20 | 118 | 0.024±0.0017 | 215 |
|  | **p value** | | **p value** | | **p value** | |
| untreated vs. healthy control | ns >0.9999 | | ns >0.9999 | | ns 0.7170 | |
| untreated vs. disease serum | ns 0.2650 | | ns >0.9999 | | *0.0288 | |
| healthy control vs. disease serum | ns 0.8641 | | ns >0.9999 | | *0.0288 | |

Significances were calculated usin7 one-way ANOVA followed by Holm-Šídák´s post hoc test or using Kruskal-Wallis followed by Dunn´s post hoc test tests for multiple comparisons.: *p≤0.05; **p≤0.01; ***p≤0.001; ****p≤0.0001; ns = not significant

**Video S1.** Neurite extension SCN, SCN-AC, SCN-AC-LN

**Video S2.** Synapse density SCN, SCN-AC, SCN-AC-LN

**Video S3.** Ca^2+^-imaging SCN, SCN-AC, SCN-AC-LN

**Video S4.** Ca^2+^-imaging healthy control vs. disease condition SCN-AC-LN
