# Supplementary figures and images for: Purpose‐Adaptable Reinforced 3D Hyaluronic‐Acid Based Platform to Study Pathomechanisms of the Central Nervous System
Source: Adv Healthc Mater. 2026 Feb 15;15(17):e05946. doi: 10.1002/adhm.202505946 (PMC13175302; doi:10.1002/adhm.202505946)

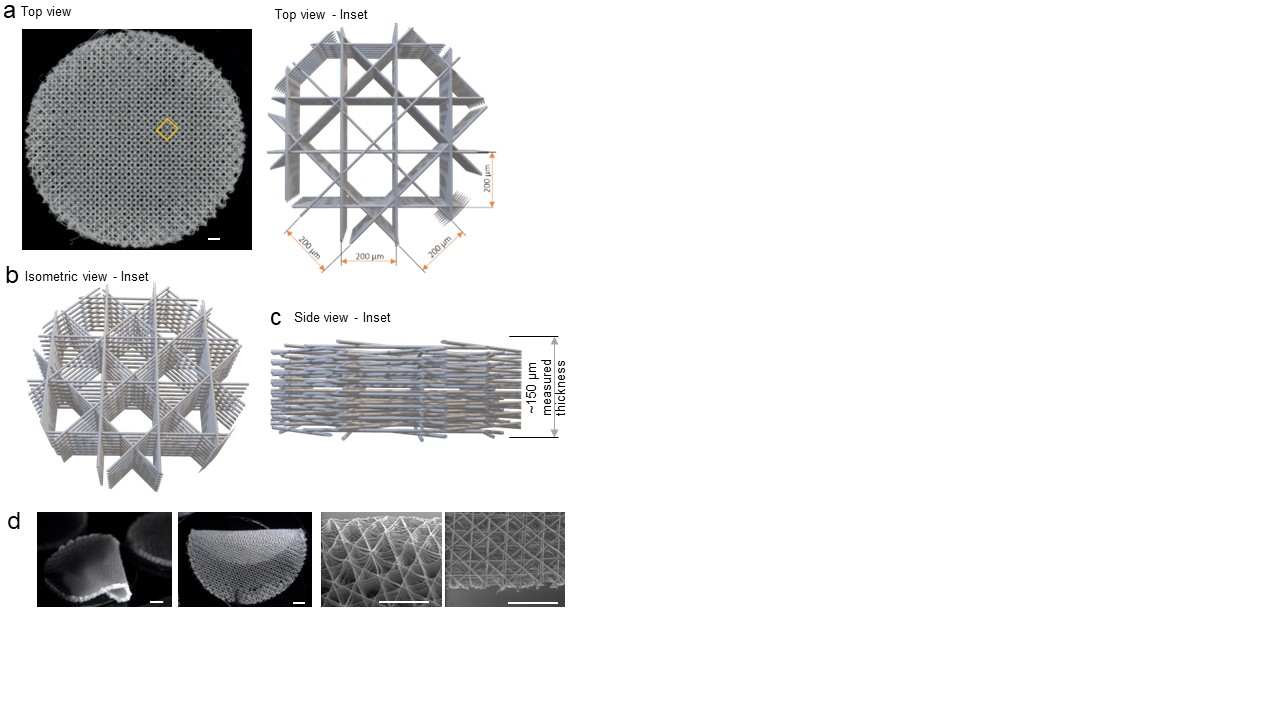

Supplement: Supplementary file 2 — Supporting File 2: adhm70940‐sup‐0002‐FigureS1‐S4.zip. [file ADHM-15-0-s001.zip › Figure_S1.jpg]

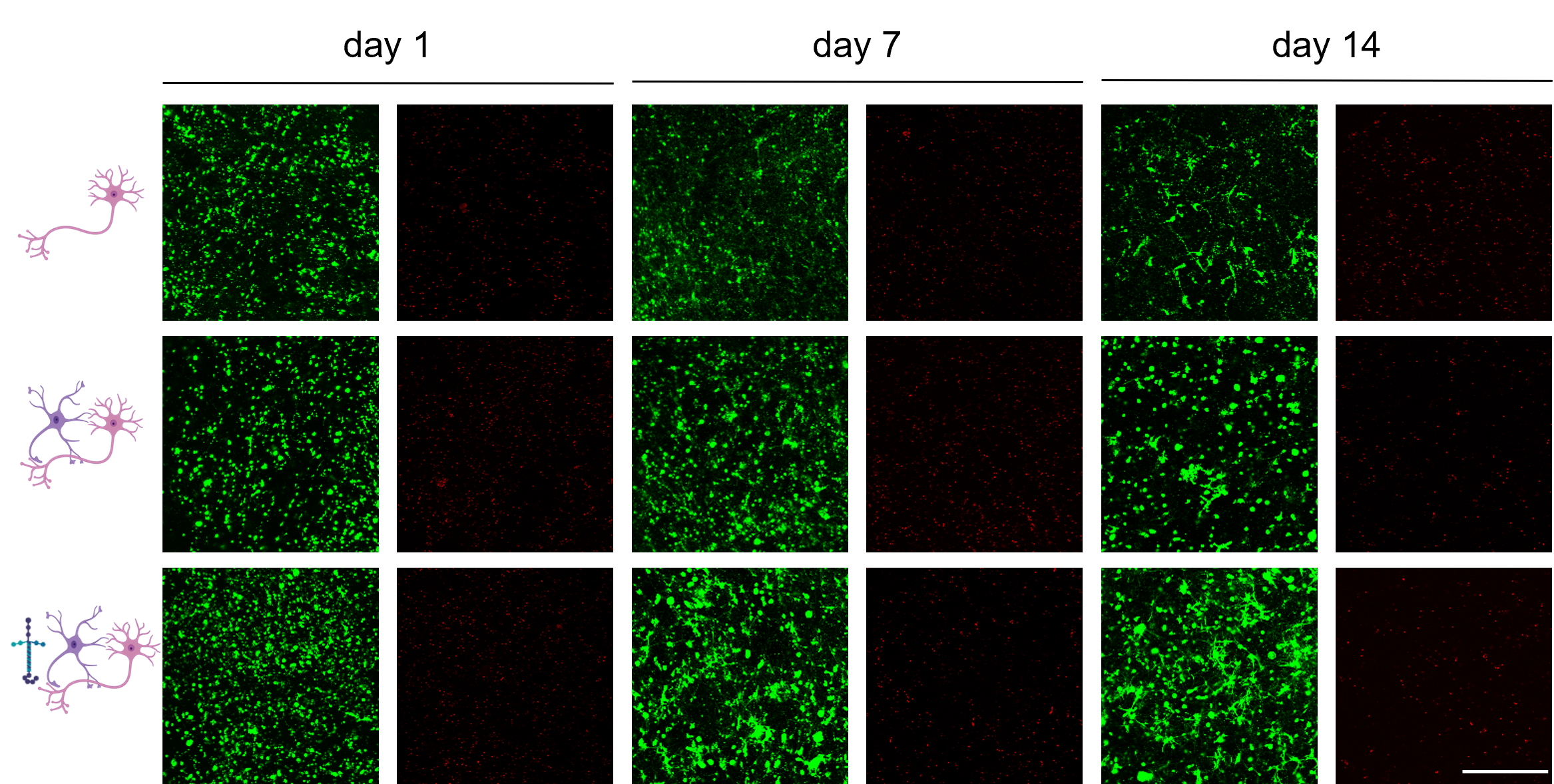

Supplement: Supplementary file 2 — Supporting File 2: adhm70940‐sup‐0002‐FigureS1‐S4.zip. [file ADHM-15-0-s001.zip › Figure_S2.png]

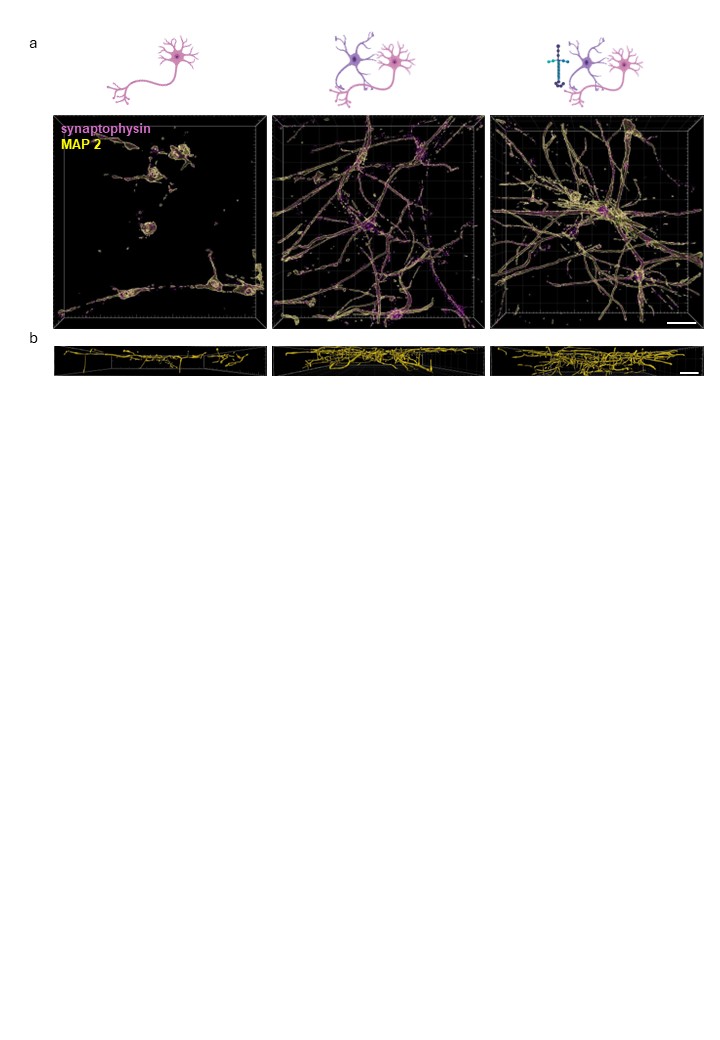

Supplement: Supplementary file 2 — Supporting File 2: adhm70940‐sup‐0002‐FigureS1‐S4.zip. [file ADHM-15-0-s001.zip › Figure_S3.JPG]
